# Differential efficacy and anti-inflammatory mechanisms of Bailing Preparations versus Huangkui Capsules combined with SGLT-2 inhibitors for diabetic kidney disease: a network meta-analysis and GRADE assessment

**DOI:** 10.3389/fphar.2026.1812118

**Published:** 2026-05-29

**Authors:** Xinyu Li, Xiao Yuan, Zhiyu Wu

**Affiliations:** 1 Department of Pharmacy, West China School of Medicine, Sichuan University, Sichuan University Affiliated Chengdu Second People’s Hospital, Chengdu Second People’s Hospital, Chengdu, Sichuan, China; 2 Department of Pharmacy, The Third Affiliated Hospital of Chengdu Medical College, Chengdu Pidu District People’s Hospital, Chengdu, Sichuan, China

**Keywords:** Bailing Preparations, diabetic kidney disease, GRADE, Huangkui Capsules, network meta-analysis, SGLT-2 inhibitors

## Abstract

**Background:**

While sodium-glucose cotransporter 2 (SGLT-2) inhibitors serve as the cornerstone of therapy for diabetic kidney disease (DKD), a specific subset of patients continues to face residual risks, manifested as persistent proteinuria or declining renal function. Bailing Preparations and Huangkui Capsules are widely employed adjunctive Commercial Chinese polyherbal preparations (CCPPs). However, rigorous data differentiating their specific efficacy profiles—particularly regarding anti-inflammatory targets when paired with SGLT-2 inhibitors—remain undefined.

**Methods:**

Computerized searches were conducted in PubMed, Embase, Cochrane Library, Web of Science, China National Knowledge Infrastructure (CNKI), WanFang Database, VIP Database, China Biomedical Literature Service System (Sinomed), and Bailing Preparation + SGLT-2i, Huangkui Capsule + SGLT-2i, and SGLT-2i monotherapy for the treatment of DKD. The search period covered from the establishment of each database to December 2025. The risk of bias was evaluated using the Cochrane RoB 2.0 tool, a network meta-analysis was performed using Stata 19.5, and the quality of evidence was assessed using the GRADE method.

**Results:**

The final analysis included 18 RCTs, involving a total of 1,564 participants. The results of the network meta-analysis showed: (1) Renal function and clinical efficacy: In terms of renal function protection, the Bailing preparation had the most significant effect in reducing serum creatinine (Scr) (MD = −12.31, 95% CI: −17.14, −7.48), and its Surface Under the Cumulative Ranking curve (SUCRA) probability ranking (81.8%) was the highest. The ranking results of the overall clinical efficacy (SUCRA = 79.1%) also supported this trend, with the Bailing preparation slightly superior to Huangkui Capsules. (2) Proteinuria and metabolism: Regarding proteinuria and metabolic indicators, Huangkui Capsules demonstrated unique advantages. In terms of 24-h urine protein quantification, Huangkui Capsules was the only intervention measure with a significant reduction effect (MD = −0.47, 95% CI: −0.77, −0.18). Additionally, its effects on fasting blood glucose (FPG) and 2-h post-meal blood glucose (2hPG) ranked the highest among all intervention measures (SUCRA values were all >90%). (3) Anti-inflammatory mechanism: The Bailing preparation specifically reduced systemic inflammatory markers (TNF-α and CRP), while Huangkui Capsules reduced local inflammatory mediators IL-6. (4) Safety: The SUCRA value of Huangkui Capsules was the highest (78.6%), indicating better safety.

**Conclusion:**

Both Bailing Preparations and Huangkui Capsules can enhance the efficacy of SGLT-2i. Bailing Preparations focuses on protecting renal function by inhibiting systemic inflammation mediated by TNFα and CRP; Huangkui Capsules focuses on regulating glucose metabolism and inhibiting local damage mediated by IL-6 to reduce proteinuria.

## 1 Introduction

With the global economic development and changes in people’s lifestyles and dietary habits, the number of people suffering from diabetic kidney disease (DKD) is increasing. DKD is the primary cause of end-stage renal disease (ESRD) (Sun et al., 2022; Tuttle et al., 2014). In recent years, sodium-glucose cotransporter 2 (SGLT-2) inhibitors have become the first-line drugs for treating DKD due to their dual effects of improving glomerular hyperfiltration and metabolic reprogramming (Zhao and Xie, 2025). Clinical studies have shown that using SGLT-2 inhibitors alone to treat DKD cannot completely stop the disease progression in all patients, especially in those with micro-inflammatory states or massive proteinuria, where residual renal risks still exist. Therefore, it is necessary to explore reasonable combined treatment strategies on this basis.

Traditional Chinese Medicine (TCM) plays a significant role in the treatment of DKD. Modern pharmacological studies have shown that various single-component extracts of traditional botanical drugs exhibit remarkable renal protective effects in the treatment of DKD. The main components of Bailing Preparations (including Bailing capsules and Bailing tablets) are fermented Cordyceps sinensis mycelia. Traditional medicine considers it to have the efficacy of “tonifying the lung and kidney,” and modern pharmacology has confirmed that it has antioxidant, anti-fibrotic and immunomodulatory effects (Liu and Wu, 2015). The main component of Huangkui Capsules is Abelmoschus manihot extract, which mainly contains flavonoids and is known for its “cooling heat and promoting diuresis” properties. It has been proven to effectively protect podocytes and reduce proteinuria (Zou et al., 2026).

Although some meta-analyses have separately investigated the efficacy of Bailing Preparations or Huangkui capsules, there is still a lack of direct head-to-head comparisons between the two, and the differences in the anti-inflammatory mechanisms when they are combined with SGLT-2 inhibitors have not been systematically elaborated. Clinicians often lack clear selection criteria when dealing with patients with different characteristics (such as those mainly characterized by elevated creatinine vs those mainly by proteinuria). Inflammation, as the core driving force of DKD progression, different factors play differentiated roles in renal injury. For example, systemic inflammatory factor TNF-α and local inflammatory factor IL-6 may mediate different pathological processes.

This study aims to indirectly compare the differences in 11 indicators between Bailing preparation and Huangkui Capsule combined with SGLT-2 inhibitors through Network Meta-Analysis (NMA). In particular, it will deeply analyze the specificity of both in anti-inflammatory targets, providing evidence-based support for the precise treatment of DKD. This study not only compares the differences in 11 clinical indicators between the two, but also will introduce the GRADE system for grading the quality of evidence for the first time, providing a high-quality evidence base for the formulation of clinical guidelines.

## 2 Materials and methods

### 2.1 Registration and reporting standards

This study is registered in PROSPERO (CRD420261281082). Reporting strictly adheres to the PRISMA-NMA (2015) statement and the Cochrane Handbook.

### 2.2 Botanical nomenclature and preparation composition

In this study, the two commercial Chinese polyherbal preparations (CCPPs) evaluated were Bailing Preparations and Huangkui Capsules. To adhere to the ConPhyMP guidelines, the botanical nomenclature has been strictly validated using the Medicinal Plant Names Services (MPNS, http://mpns.kew.org). Bailing Preparations are formulated from the fermented mycelia of *Ophiocordyceps sinensis* (Berk.) G.H.Sung et al. [Ophiocordycipitaceae; Ophiocordyceps sinensis mycelia]. Huangkui Capsules consist of the extract from the corolla of *Abelmoschus manihot* (L.) Medik [Malvaceae; Abelmoschi corolla]. Bailing Preparations and Huangkui Capsules are manufactured under national quality control standards in China. Bailing Capsule is monographed in the Chinese Pharmacopoeia (ChP, 2020 Edition, Part I), whereas Bailing Tablet and Huangkui Capsules are regulated under the National Drug Standards of China. The raw materials, including fermented Cordyceps sinensis powder for Bailing Preparations and Abelmoschus manihot flower for Huangkui Capsules, are standardized according to relevant Chinese regulatory requirements. The detailed composition, parts used, type of extract, and daily dosage of these CCPPs are summarized in Table 1.

**TABLE 1:** Composition and characteristics of the evaluated commercial Chinese polyherbal preparations (CCPPs).

Characteristics	Bailing Preparations (capsules/Tablets)	Huangkui Capsules
Full botanical name (Authority) [family]	*Ophiocordyceps sinensis* (Berk.) G.H.Sung et al. [Ophiocordycipitaceae]	*Abelmoschus manihot* (L.) medik. [Malvaceae]
Plant part used	Fermented mycelia	Corolla
Type of extract/Preparation	Dried powder of fermented mycelia (fermented *Cordyceps sinensi*s)	Ethanol extract of dried corolla
Quality control standard	ChP 2020, Part I (for Bailing Capsule); National Drug Standard of China (for Bailing Tablet)	National Drug Standard of China; raw material Abelmoschus manihot flower regulated under ChP 2020, Part I
Major known Active constituents	Adenosine, cordycepin, mannitol, ergosterol	Total flavonoids (e.g., hyperoside, quercetin)
Standard daily dosage in included RCTs	3.0–6.7 g/day (divided into 3 doses)	3.0g–7.5 g/day (divided into 3 doses)

### 2.3 Inclusion and exclusion criteria

#### 2.3.1 Inclusion criteria

1. Study Type: This study included randomized controlled trials (RCTs). Languages were Chinese and English.
2. Study Population: Patients diagnosed with diabetic kidney disease (DKD) with comparable baseline renal function and blood glucose levels.
3. Interventions: Group A: Bailing Preparations (including Bailing capsules or tablets) combined with SGLT-2 inhibitors. Group B: Huangkui Capsules combined with SGLT-2 inhibitors. Group C: Monotherapy with SGLT-2 inhibitors (or combined with conventional hypoglycemic and antihypertensive therapy).
4. Outcome Measures: At least one of the following: clinical efficacy rate, serum creatinine (Scr), blood urea nitrogen (BUN), 24-h urine protein (24 h-UTP), glycated hemoglobin (HbA1c), fasting plasma glucose (FPG), 2-h postprandial glucose (2hPG), inflammatory markers (TNF-α, CRP, IL-6), and adverse reactions.

#### 2.3.2 Exclusion criteria

Non-RCT studies, reviews, animal experiments, or case reports. Studies with incomplete data reporting or obvious errors that preclude meta-analysis. Duplicate publications or studies suspected of data overlap. Studies combining other Chinese botanical drug formulations with known renal effects.

### 2.4 Search strategy

Two researchers independently searched PubMed, Embase, Cochrane Library, Web of Science, and Chinese databases including China National Knowledge Infrastructure (CNKI), Wanfang Database, VIP Database, and Sinomed. The search period was from database inception to December 2025. Search terms included: “Diabetic Nephropathies,” “Diabetic Kidney Disease,” “SGLT-2 inhibitors,” “Dapagliflozin,” “Empagliflozin,” “Canagliflozin,” “Bailing,” “Corbrin,” “Huangkui,” “Abelmoschus manihot,” and others.

### 2.5 Literature screening and data extraction

Two researchers used EndNote 21.3 software to locate and remove duplicate publications, independently conducting literature screening. Disagreements were resolved through discussion; if consensus could not be reached, a third researcher assisted in decision-making. The following data were extracted from included studies: first author, publication year, number of patients, patient age, treatment regimen, treatment duration, and outcome measures.

### 2.6 Risk of bias assessment

Two researchers independently assessed the risk of bias in included studies using the Cochrane Risk of Bias tool (RoB 2.0). Domains assessed included: random sequence generation, allocation concealment, blinding (participants/researchers), assessment of outcome blinding, incomplete outcome data, selective reporting, and other biases. Each domain was rated as “low risk,” “high risk,” or “unclear.” This process evaluated the quality of included studies. Two researchers performed this assessment independently and cross-checked results; when consensus could not be reached, a third researcher assisted in reaching a judgment.

### 2.7 Statistical analysis

Data analysis was performed using the mvmeta and network packages in Stata 19.5 software.

Network Model: Mixed comparisons were performed using a frequency-based random-effects model.

Effect Size Calculation: For dichotomous variables (efficacy rate, adverse reactions), the relative risk (RR) and its 95% confidence interval (CI) were used; for continuous variables, the mean difference (MD) and its 95% CI were used.

Transitivity and Consistency Assessment: The assumption of transitivity was evaluated by comparing the distribution of potential effect modifiers across the different treatment comparisons, including participant demographics (age, baseline renal function), drug dosages, and treatment durations. The clinical and methodological characteristics were found to be sufficiently similar to justify indirect comparisons. Regarding consistency, as the network structure formed a star-shaped configuration centered on SGLT-2 inhibitors with no closed loops, the global inconsistency and node-splitting tests were not applicable. Instead, consistency was ensured through the rigorous implementation of the transitivity assumption and the use of a common random-effects model for all indirect estimates. The detailed analysis report, including the Stata data processing scripts and results of the transitivity assessment, is provided in the Supplementary Material.

Ranking analysis: SUCRA was calculated to predict the probability of each intervention becoming the optimal strategy. Higher SUCRA values (approaching 100%) indicate superior efficacy.

Publication Bias: Assessment of publication bias risk for primary outcomes using a comparison-adjusted funnel plot.

### 2.8 Evidence quality grading (GRADE assessment)

The GRADE (Grading of Recommendations Assessment, Development and Evaluation) system was used to grade the evidence from network meta-analyses for all outcome measures. Evidence quality was rated as “high,” “moderate,” “low,” or “very low” based on five downgrading factors: limitations, inconsistency, indirectness, imprecision, and publication bias.

## 3 Results

### 3.1 Literature screening process and characteristics of included studies

**FIGURE 1:**
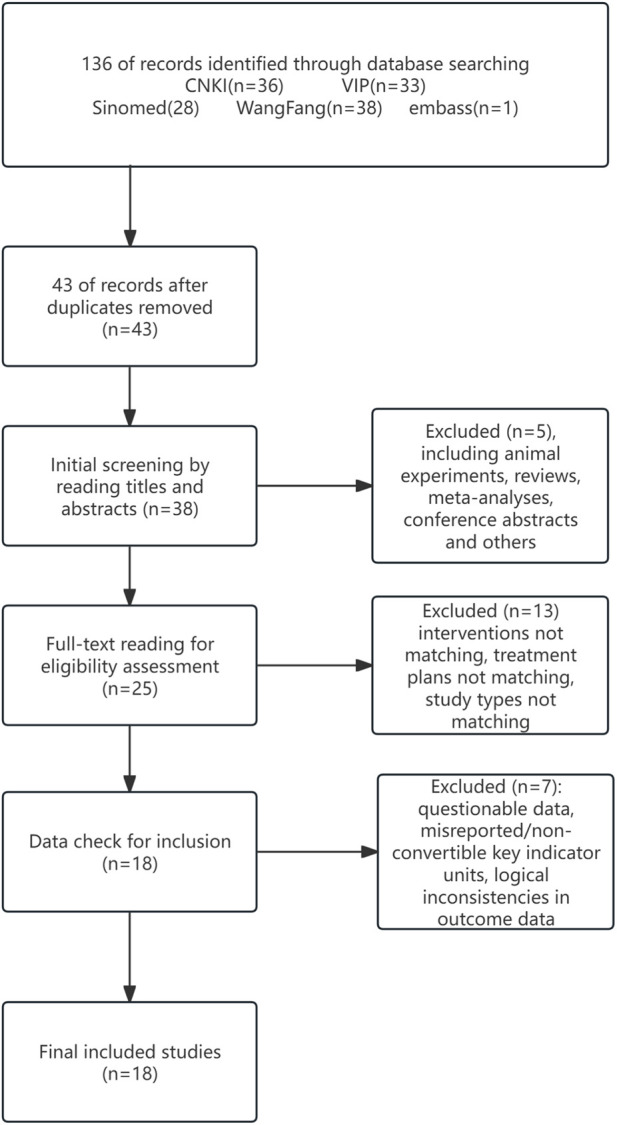
PRISMA flow diagram.

**FIGURE 2:**
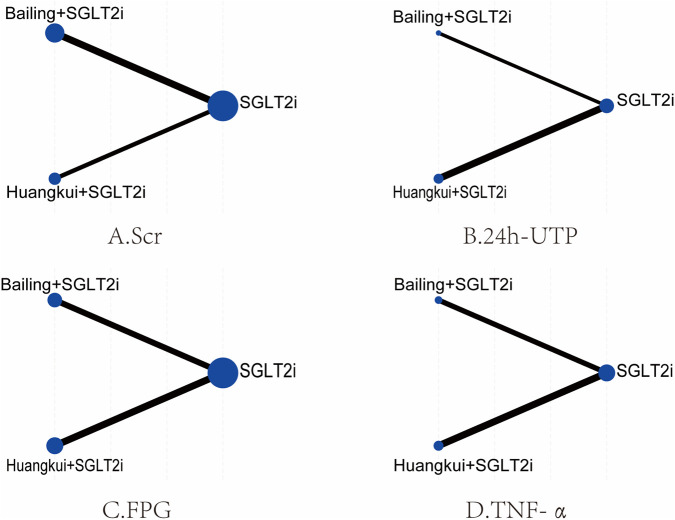
Network diagram.

The initial search yielded 136 studies. After deduplication and full-text screening, 118 studies were excluded, resulting in 18 (Jiang et al., 2023; Meng et al., 2024; Wang M. et al., 2023; Li and Gao, 2021; Sun, 2023; Dang and Zhu, 2022; Zhang and Zheng, 2024; YANG et al., 2023; Chen and Zhang, 2025; Wen and Liu, 2025; Wang and Hua, 2020; Tan, 2024; Chen et al., 2024; Zhang, 2025; Yang, 2022; Fu and Hu, 2023; Zhang et al., 2021; Zhao and Zhang, 2025) RCTs involving 1,564 patients. The literature screening flowchart is shown in Figure 1. Among these, 10 studies compared “Bailing + SGLT2i vs SGLT2i,” and 8 studies compared “Huangkui + SGLT2i vs SGLT2i.” All studies were conducted in China with balanced baseline characteristics. Seventeen studies were two-arm trials, while only Yang Deyu’s study was a three-arm trial. We selected two arms from this study, ultimately forming a star-shaped network structure centered on SGLT-2i (Figure 2). Basic characteristics of the included studies are summarized in Table 2.

**TABLE 2:** Characteristics of included studies.

First author	Year	Sample size (T/C)	Mean age (years, T/C)	SGLT-2 inhibitor	TCM intervention	Course of treatment	Outcomes
Bailing Preparations + SGLT-2i vs. SGLT-2i							
Fu wei	2023	39/39	68.9/69.4	Ertugliflozin	Bailing capsule	3 months	1,2,3,4,5,6,7,11
Li xiaoqin	2021	58/58	55.2/55.7	Dapagliflozin	Bailing capsule	3 months	2,10,11
Wen jing	2025	38/38	NR	Dapagliflozin	Bailing capsule	3 months	1,2,3,8,9,10
Wang mei	2023	40/40	63.3/63.4	Dapagliflozin	Bailing capsule	3 months	1,2,5,6,7,8,9,11
Zhang shaoxin	2024	52/53	58.7/58.3	Dapagliflozin	Bailing tablet	2 months	1,2,3,5,6,11
Zhang weijie	2021	35/35	56.2/55.1	Dapagliflozin	Bailing tablet	2 months	1,3,5,7,11
Dang qian	2022	42/43	64.1/64.9	Dapagliflozin	Bailing capsule	3 months	2, 4, 8, 11
Chen Li	2025	51/51	55.5/55.6	Dapagliflozin	Bailing capsule	3 months	1,2,3,9,10,11
Meng yanping	2024	51/51	59.1/59.1	Dapagliflozin	Bailing capsule	2 months	1,2,3,5,6,7
Zhao xiaorong	2025	34/33	53.2/53.1	Dapagliflozin	Bailing capsule	3 months	1,3,5,6,11
Huangkui capsule + SGLT-2i vs. SGLT-2i							
Yang deyu	2023	42/35	65.2/64.8	Dapagliflozin	Huangkui capsule	3 months	5,6,7
Yang Yue	2022	48/48	47.6/48.5	Dapagliflozin	Huangkui capsule	NR	2,5,6,7,11
Wang Baofeng	2020	30/30	52.6/51.7	Dapagliflozin	Huangkui capsule	3 months	1,4,5,6,7,11
Zhang xiujuan	2025	40/40	53.8/54.1	Dapagliflozin	Huangkui capsule	3 months	2, 3, 5, 6, 7, 8, 10, 11
Jiang meiqiong	2023	44/44	66.5/66.9	Dapagliflozin	Huangkui capsule	4 months	1,2,3,4,5,6,7,8,9,10,11
Sun ying	2023	43/43	65.5/64.2	Empagliflozin	Huangkui capsule	2 months	1,2,3,9,10
Chen yujie	2024	48/48	64.3/64.2	Dapagliflozin	Huangkui capsule	3 months	1,2,3,4,5,6,7,8,9,10,11
Tan jie	2024	50/50	67.3/67.1	Dapagliflozin	Huangkui capsule	3 months	1, 4, 5, 6, 7, 8, 9, 10, 11

T, Treatment group; C, Control group; NR, Not reported; TCM, Traditional Chinese Medicine; SGLT-2i, Sodium-glucose cotransporter-2 inhibitor. Outcomes: (1) Clinical effective rate; (2) Scr (Serum creatinine); (3) BUN (Blood urea nitrogen);(4) 24h-UTP (24-hour urinary total protein); (5) FPG (Fasting plasma glucose);(6) 2hPG (2-hour postprandial glucose); (7) HbA1c (Glycosylated hemoglobin); (8) TNF-α (Tumor necrosis factor-alpha); (9) CRP (C-reactive protein); (10) IL-6 (Interleukin-6); (11) Adverse reactions.

During data extraction and cleaning, we identified the following issues in some reports, excluding them from the final quantitative synthesis (network meta-analysis): 1. Critical indicators reported in incorrect units or unable to be converted: For example, Fang and Hu (2022) reported serum creatinine units as ‘mmol/L’, which does not match the standard clinical reporting unit ‘μmol/L’. Furthermore, after unit conversion, the numerical magnitude is entirely inconsistent with clinical values. 2. Logical inconsistencies in results data: For instance, studies by Zhu and Qiu (2025) and Wang et al. (2025) reported abnormal, substantial increases in eGFR (exceeding 30 mL/min/1.73 m^2^) following treatment. This sharply contradicts the expected physiological response to diabetic nephropathy therapy, casting doubt on data reliability. A total of 7 studies were excluded.

Following this rigorous screening process, 18 studies ultimately provided consistent, high-quality data suitable for quantitative synthesis.

### 3.2 Risk of bias assessment results

**FIGURE 3:**
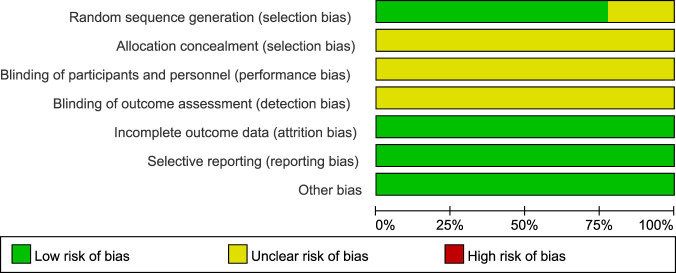
Cochrane risk of bias graph.

**FIGURE 4:**
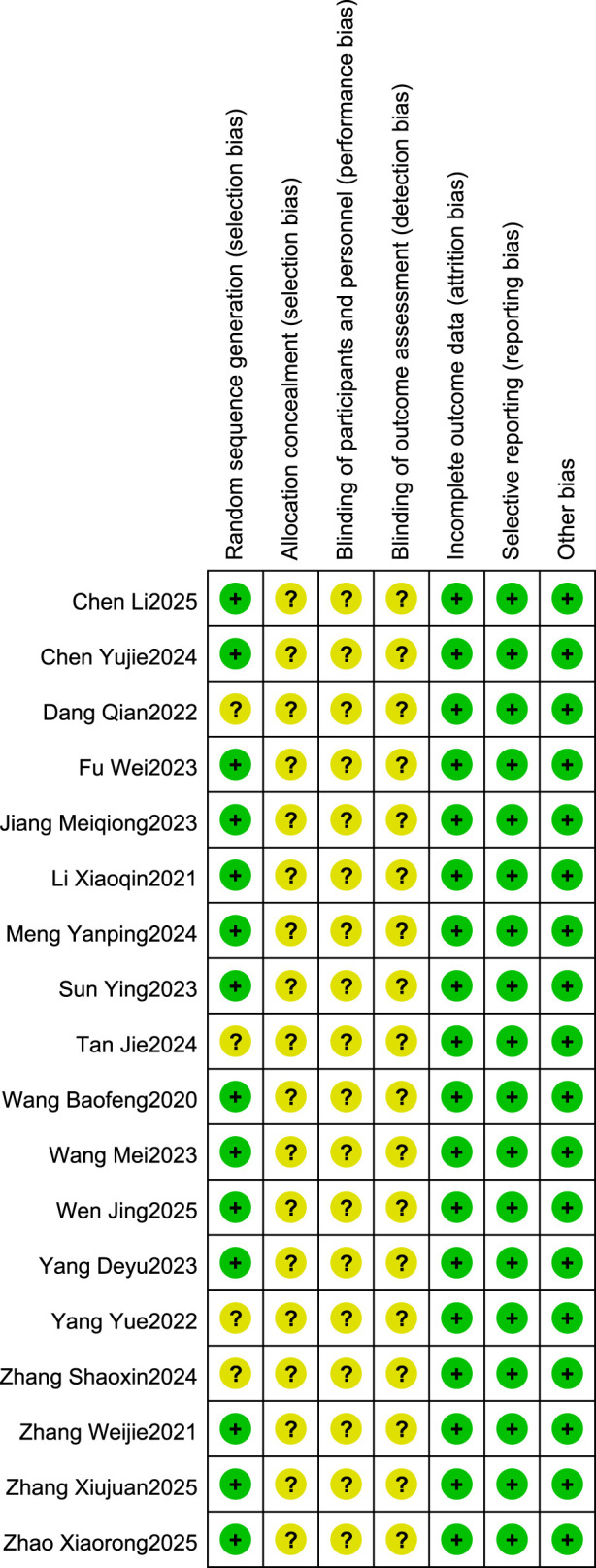
Cochrane risk of bias summary.

Cochrane RoB 2.0 assessment indicated low risk for most studies. Fourteen included studies were rated as low risk for “random sequence generation” (mentioning “random number table method”), while the remaining studies did not specify randomization methods and were rated as “unclear.” Due to the visual differences between traditional Chinese medicine formulations and Biomedicine, none of the studies mentioned double-blinding; thus, “blinding” and “allocation concealment” were rated as “unclear” or “high risk.” All studies reported complete outcome data and showed no evidence of selective reporting, thus rated as “low risk.” Overall, the methodological quality of the included studies was acceptable. Results are presented in Figures 3, 4.

### 3.3 Heterogeneity and consistency analysis

Analysis using a random-effects model revealed that the global heterogeneity parameters ( $\tau^{2}$ ) for all outcome measures in this study were generally low, indicating good methodological and clinical homogeneity among the included studies and robust statistical pooling results. Furthermore, since the network evidence structure constructed in this study exhibits a “star-shaped” configuration centered on SGLT-2 inhibitors, and there are no direct comparative RCTs between the Bailing Preparations group and the Huangkui Capsules group (zero closed loops), neither node-splitting methods nor inconsistency tests are required. The effect sizes for both drug comparisons in this study were estimated indirectly based on the transitivity assumption.

### 3.4 Renal function and clinical effective rate

**FIGURE 5:**
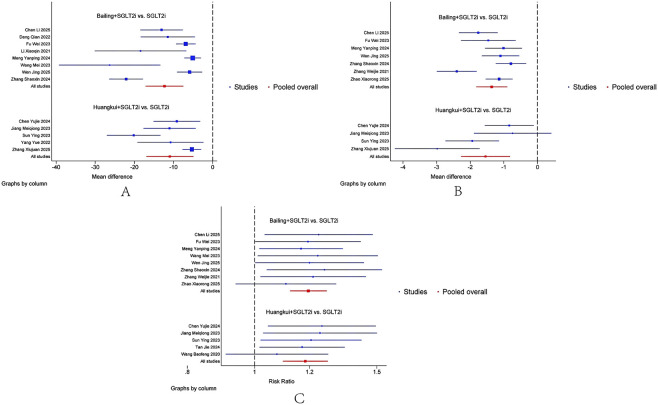
Forest plot of Renal Function and Clinical Effective Rate. **(A)** Scr. **(B)** BUN. **(C)** Clinical effective rate.

**FIGURE 6:**
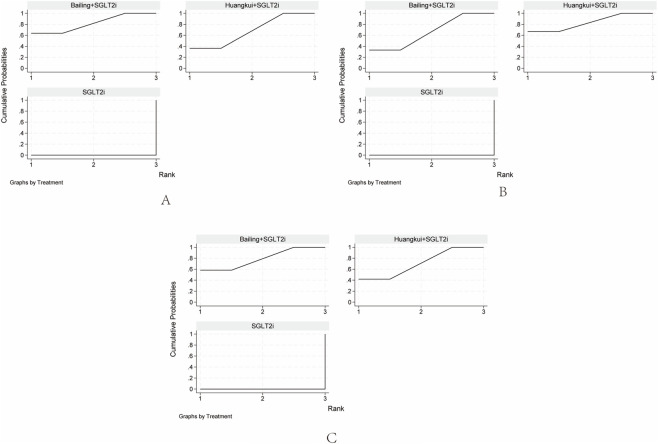
SUCRA of renal function & clinical effective rate. **(A)** Scr. **(B)** BUN. **(C)** Clinical effective rate.

**FIGURE 7:**
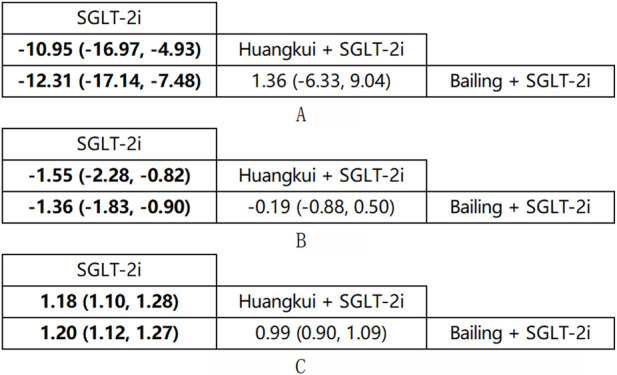
Network League Table of Renal Function & Clinical Effective Rate. Note: Bold text indicates statistically significant differences (the 95% confidence interval does not cross the null line of 0 for MD or 1 for RR). **(A)** Scr. **(B)** BUN. **(C)** Clinical effective rate.

The results of the network meta-analysis indicate that both combinations of Commercial Chinese polyherbal preparations (CCPPs) can improve renal function, albeit to varying degrees, as shown in Figures 5–7.

Serum creatinine (Scr): Compared with SGLT-2 inhibitors monotherapy, both the Bailing Preparations group (MD = −12.31, 95% CI: −17.14, −7.48) and the Huangkui Capsules group (MD = −10.95, 95% CI: −16.97, −4.93) significantly reduced serum creatinine levels. Although the network comparison between the two drugs did not reach statistical significance, the SUCRA ranking placed Bailing Preparations (81.8%) first, indicating its best potential for protecting glomerular filtration function.

Blood Urea Nitrogen (BUN):Compared to SGLT-2i monotherapy, both the Bailing Preparations group (MD = −1.36, 95% CI: −1.83, −0.90) and the Huangkui Capsules group (MD = −1.55, 95% CI: −2.28, −0.82) significantly reduced blood urea nitrogen levels. Notably, although the network comparison between the two drugs did not reach statistical significance, the Huangkui Capsules group (SUCRA 83.4%) ranked higher than the Bailing Preparations group (SUCRA 66.6%), suggesting Huangkui may have a relative advantage in promoting the clearance of nitrogenous metabolic products.

Overall clinical response rate: Both the Bailing Preparations group (RR = 1.20, 95% CI: 1.12, 1.27) and the Huangkui Capsules group (RR = 1.18, CI: 1.10, 1.28) demonstrated significantly higher overall response rates than the monotherapy group. The two drugs showed comparable efficacy, with the SUCRA score for Bailing Preparations (79.1%) slightly higher than that for Huangkui Capsules (70.9%).

### 3.5 Proteinuria and glucose metabolism

24-h Urinary Protein (24 h-UTP): Huangkui Capsules were the only intervention showing a statistically significant difference for this outcome (MD = −0.47, 95% CI: −0.77, −0.18). In contrast, the difference between the Bailing Preparations group and the monotherapy group was not statistically significant (MD = −0.08, 95% CI: −0.47, 0.32). This indicates that Huangkui Capsules are superior to Bailing Preparations in reducing proteinuria.

**FIGURE 8:**
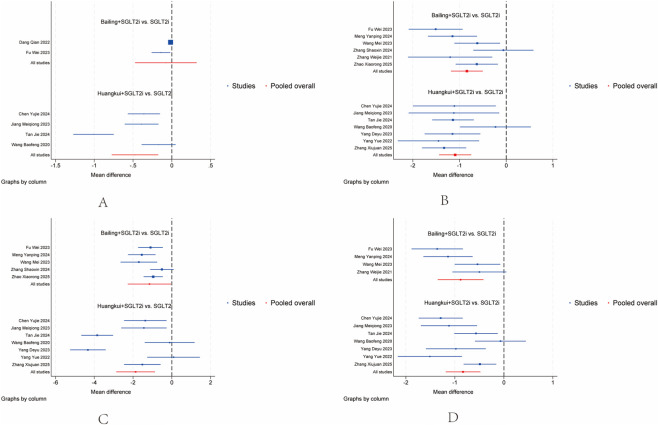
Forest plot of Proteinuria & Glucose Metabolism. **(A)** 24h-UTP. **(B)** FPG. **(C)** 2hPG. **(D)** HbAlc.

**FIGURE 9:**
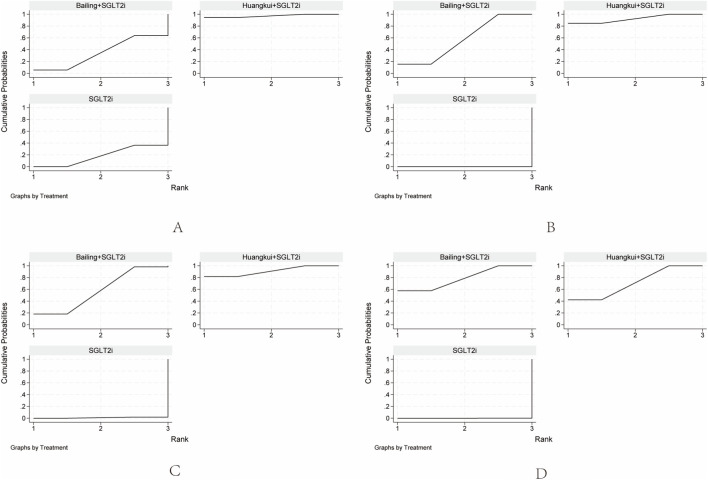
SUCRA of proteinuria & glucose metabolism. **(A)** 24h-UTP. **(B)** FPG. **(C)** 2hPG. **(D)** HbAlc.

**FIGURE 10:**
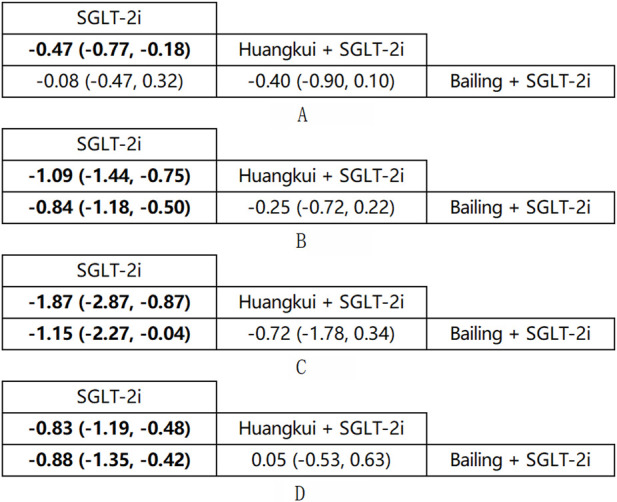
Network League Table of Proteinuria & Glucose Metabolism. Note: Bold text indicates statistically significant differences (the 95% confidence interval does not cross the null line of 0 for MD or 1 for RR). **(A)** 24h-UTP. **(B)** FPG. **(C)** 2hPG. **(D)** HbAlc.

Glucose metabolism (FPG, 2hPG, HbA1c): Both drugs demonstrated efficacy in lowering fasting plasma glucose (FPG) and 2-h postprandial glucose (2hPG). However, Huangkui Capsules ranked first in SUCRA rankings for both indicators (92.3% and 90.8%, respectively) and achieved greater mean reductions than Bailing Preparations, suggesting stronger synergistic hypoglycemic effects when combined with SGLT-2 inhibitors. Regarding glycated hemoglobin (HbA1c), both drugs demonstrated comparable efficacy, with Bailing Preparations achieving a SUCRA ranking of 78.8% and Huangkui Capsules achieving 71.2%. See Figures 8–10.

### 3.6 Divergence in inflammatory targets

**FIGURE 11:**
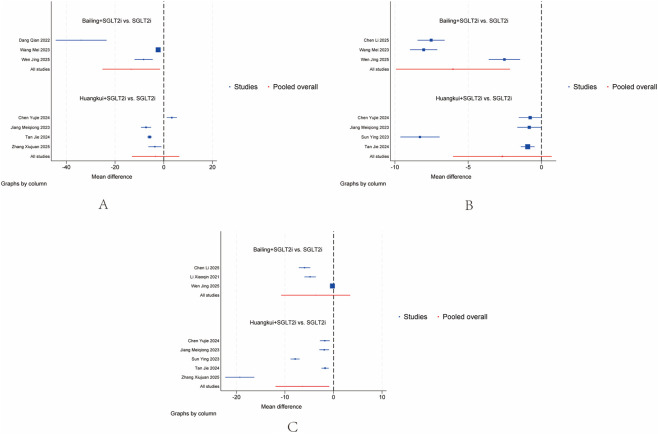
Forest plot of inflammatory factors. **(A)** TNF-α. **(B)** CRP. **(C)** IL-6.

**FIGURE 12:**
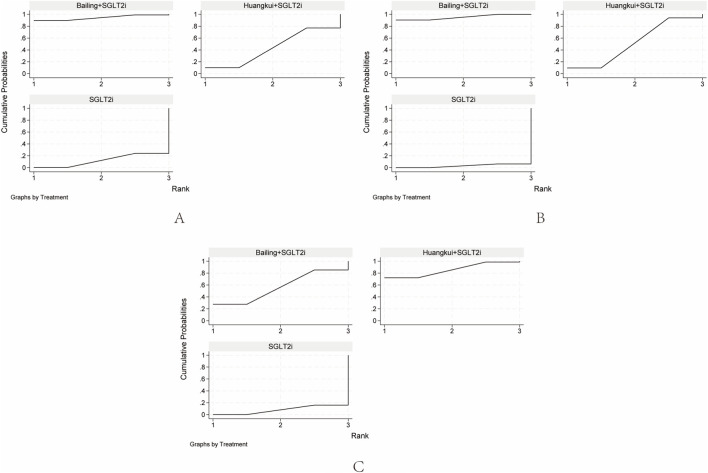
SUCRA of inflammatory factors. **(A)** TNF-α. **(B)** CRP. **(C)** IL-6.

**FIGURE 13:**
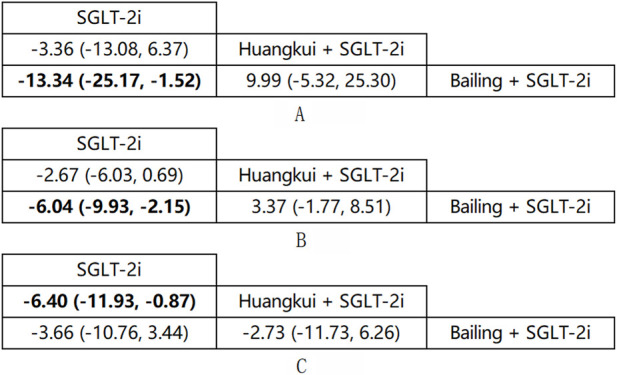
Network League Table of inflammatory factors. Note: Bold text indicates statistically significant differences (the 95% confidence interval does not cross the null line of 0 for MD or 1 for RR). **(A)** TNF-α. **(B)** CRP. **(C)** IL-6.

This study revealed a significant “dual divergence” phenomenon in anti-inflammatory targets through network meta-analysis, as shown in Figures 11–13:

Systemic Inflammation (TNF-α & CRP): Bailing Preparations specifically reduced systemic inflammatory factors. It significantly reduced TNF-α (MD = −13.34, 95% CI: −25.17, −1.52) and CRP (MD = -6.04, 95% CI: −9.93, −2.15) levels. In contrast, Huangkui Capsules showed no statistically significant difference compared to the monotherapy group for either of these markers.

Local/Mesangial Inflammation (IL-6): Conversely, Huangkui Capsules specifically and significantly reduced IL-6 levels (MD = −6.40, 95% CI: −11.93, −0.87), while Bailing Preparations had no significant effect on this marker. Given that IL-6 is closely associated with glomerular mesangial proliferation and podocyte injury, this finding is highly consistent with the superior protein-lowering effect of Huangkui.

### 3.7 Safety and publication bias

**FIGURE 14:**
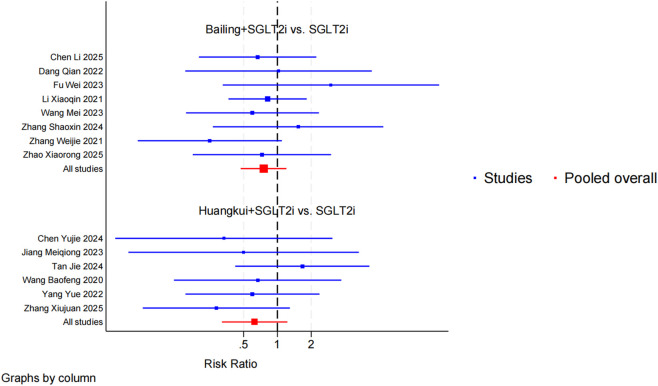
Forest plot of Adverse Reactions.

**FIGURE 15:**
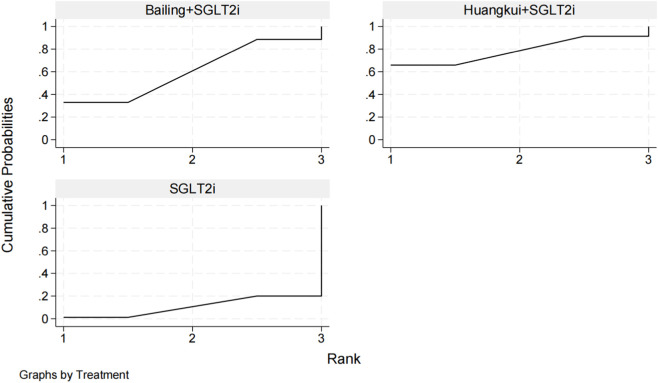
SUCRA of adverse reactions.

Regarding adverse reactions (e.g., urinary tract infections, hypoglycemia, gastrointestinal reactions), although no statistically significant differences were observed between the combination therapy groups and the SGLT-2i monotherapy group (all 95% CIs spanned 1), point estimates indicated a trend toward reduced adverse reaction incidence (Bailing RR = 0.75; Huangkui RR = 0.63). SUCRA ranking indicated the highest probability of safety for the Huangkui Capsules combination group (78.6%), followed by Bailing Preparations (60.8%), suggesting combination therapy did not increase and may even reduce disease progression-related adverse events. Publication bias analysis: Publication bias risk was assessed by plotting funnel plots for each outcome measure. Results showed that funnel plots for overall response rate, SCR, BUN, and glucose-related indicators exhibited essentially symmetrical shapes with scattered points evenly distributed on both sides of the vertical line, indicating no significant publication bias. For indicators with fewer included studies, such as inflammatory factors and 24 h UTP, funnel plots also showed no obvious asymmetric distribution patterns. Overall, the study results are robust, and the risk of publication bias is manageable. See Figures 14, 15.

### 3.8 GRADE evidence quality grading results

Among the 11 outcome measures, the quality of evidence for 6 outcomes was rated as “Moderate,” while 5 outcomes were rated as “Low.” The primary reasons for downgrading were risks of bias due to potential issues with blinding implementation in some studies, as well as small sample sizes and wide confidence intervals (imprecision). See Table 3.

**TABLE 3:** GRADE evidence quality grading results.

Key outcomes	Relative effect (95% CI) compared with SGLT-2i monotherapy	Number of studies	Evidence quality (GRADE)	Conclusions and interpretation
Total effective rate (total effective Rate)	Bailing: RR 1.20 (1.12, 1.27) Huangkui: RR 1.18 (1.10, 1.28)	13	⊕⊕⊕○ moderate	Both groups significantly outperformed monotherapy with comparable efficacy. Bailing ranked slightly higher.
Serum creatinine (SCR)	Bailing: MD -12.31 (−17.14, −7.48) Huangkui: MD -10.95 (−16.97, −4.93)	13	⊕⊕⊕○ moderate^a^	Both groups significantly reduced SCR. The Bailing group showed a slightly greater mean reduction and ranked first.
Blood urea nitrogen (BUN)	Bailing: MD -1.36 (−1.83, −0.90) Huangkui: MD -1.55 (−2.28, −0.82)	11	⊕⊕⊕○ moderate^a^	Both groups significantly reduced BUN, with the huangkui group showing a greater reduction and ranking first.
24-h urinary protein (24 h UTP)	Bailing: MD -0.08 (−0.47, 0.32) Huangkui: MD -0.47 (−0.77, −0.18)	6	⊕⊕○○ low^a^,^b^	Only the huangkui group showed a significant reduction in proteinuria; Bailing Preparations showed no significant advantage in reducing proteinuria compared to monotherapy
Glycated hemoglobin (HbA1c)	Bailing: MD -0.88 (−1.35, −0.42) Huangkui: MD -0.83 (−1.19, −0.48)	11	⊕⊕⊕○ moderate	Both groups significantly reduced HbA1c with comparable efficacy.
Fasting blood glucose (FPG)	Bailing: MD -0.84 (−1.18, −0.50) Huangkui: MD -1.09 (−1.44, −0.75)	13	⊕⊕⊕○ moderate^a^	Both groups showed significant reductions in FPG. The average reduction was slightly greater in the huangkui group.
2-h postprandial glucose (2HPG)	Bailing: MD -1.15 (−2.27, −0.04) Huangkui: MD -1.87 (−2.87, −0.87)	12	⊕⊕⊕○ moderate^a^	Both groups showed significant reduction in 2HPG. The average reduction was slightly greater in the huangkui group.
Adverse reactions (adverse Reactions)	Bailing: RR 0.75 (0.47, 1.20) Huangkui: RR 0.63 (0.32, 1.23)	14	⊕⊕○○ low^a^,^b^	Both groups showed a trend toward reduced adverse reactions, but the difference did not reach statistical significance. The huangkui group ranked first in safety.
TNF-α	Bailing: MD -13.34 (−25.17, −1.52)* Huangkui: MD -3.36 (−13.08, 6.37)	7	⊕⊕○○ low^a^,^b^	Bailing specifically inhibits TNF-α; huangkui has no significant effect.
CRP (C-reactive protein)	Bailing: MD -6.04 (−9.93, −2.15)* Huangkui: MD -2.67 (−6.03, 0.69)	7	⊕⊕○○ low^a^,^b^	Bailing specifically reduces CRP; huangkui shows no significant effect.
IL-6 (Interleukin-6)	Bailing: MD -3.66 (−10.76, 3.44) Huangkui: MD -6.40 (−11.93, −0.87)*	8	⊕⊕○○ low^a^,^b^	Huangkui specifically inhibits IL-6; Bailing shows no significant effect.

a Limitations: Downgraded one level. Most included studies did not clearly report allocation concealment and blinding (Unclear risk of bias).

b Imprecision: Downgraded one level. Due to small sample sizes and wide confidence intervals (spanning the null line 1.0 or 0).

## 4 Discussion

### 4.1 Key findings: dual dissociation

This study is the first GRADE-based network meta-analysis to systematically reveal a “dual separation” effect of Bailing Preparations and Huangkui Capsules in DKD treatment: Bailing Preparations excel in renal protection (Scr) and systemic anti-inflammation, while Huangkui Capsules excel in protein reduction (24 h-UTP) and local anti-inflammation. This finding challenges the previous notion of “homogeneous efficacy among Commercial Chinese polyherbal preparations (CCPPs).”

### 4.2 Bailing Preparations: systemic immunomodulator

The primary component of Bailing Preparations is Cordyceps sinensis powder. Cordyceps sinensis is a dried composite of the fruiting body and larval carcass of the fungus Cordyceps sinensis (BerK.) Sacc. parasitizing the larvae of Lepidoptera moths. It has a sweet taste and warm nature, entering the lung and kidney meridians. Its primary constituents include adenosine, cordycepin, and ergosterol. Ancient pharmacopoeias such as Compendium of Materia Medica Supplement, Essentials of Materia Medica, and Examination of Drug Properties document its efficacy in tonifying the lungs and kidneys, arresting bleeding, and resolving phlegm (Zhang et al., 2024).

Our results show that the Bailing preparation is more effective in reducing serum creatinine and systemic inflammatory factors (TNF-α, CRP). Existing studies have indicated that TNF-α is a key factor driving the systemic micro-inflammatory state and renal interstitial fibrosis in DKD (Wang Y. et al., 2023). Therefore, we hypothesize that the Bailing preparation may delay renal tubular atrophy and interstitial fibrosis by inhibiting the TNF-α signaling pathway and reducing the deposition of extracellular matrix (ECM). This inference is in perfect agreement with our analysis results.

### 4.3 Huangkui Capsules: podocyte and metabolic protector

Huangkui Capsules consist of the extract from the dried corolla of Abelmoschus manihot (L.) Medicus, a medicinal plant belonging to the Malvaceae family. The Compendium of Materia Medica records its sweet taste and cold nature, entering the kidney and bladder meridians. It clears damp-heat, reduces swelling, and detoxifies, primarily treating damp-heat sores and damp-induced edema, with extensive clinical applications (Wei et al., 2023).

Our results show that Huangkui Capsule is more effective in reducing proteinuria and lowering blood sugar, and it can specifically reduce IL-6. When IL-6 is highly expressed in all types of kidney cells, it leads to endothelial changes, podocyte damage, glomerular hypertrophy, and increased expression of mesangial fibronectin (Kreiner et al., 2022). Therefore, IL-6 is widely regarded as an important factor accelerating the progression of DKD. Our research results precisely align with this mechanism: Huangkui Capsule not only significantly reduces IL-6 but also significantly reduces 24 h-UTP at the same time, and the two show a high degree of effect consistency. The reduction of blood sugar by Huangkui Capsule may be related to its improvement of insulin resistance and regulation of oxidative stress. Therefore, Huangkui Capsule not only can control diabetic kidney disease (DKD) itself but also brings benefits to the fundamental cause of DKD (high blood sugar).

### 4.4 Synergistic efficacy and toxicity reduction of combination therapy

SGLT-2 inhibitors, while highly effective, are clinically associated with specific adverse events, notably urogenital infections. Our safety analysis revealed that combining SGLT-2i with CCPPs did not exacerbate these risks, but rather showed a trend towards reducing overall adverse events (RR < 1). From a mechanistic perspective, SGLT-2i promotes continuous glucose excretion, which in traditional Chinese medicine theory can exacerbate “Yin deficiency.” Bailing Preparations act to nourish lung and kidney Yin, while Huangkui Capsules clear damp-heat and promote diuresis. The synergistic application of these CCPPs effectively neutralizes the potential “dryness” or “damp-heat” induced by SGLT-2i, clinically manifesting as “toxicity reduction” (attenuation of side effects). Furthermore, both Bailing and Huangkui preparations have extensive histories of clinical application with well-documented general safety profiles, rarely inducing severe hepatic or renal toxicity independently. Therefore, the combination therapy not only enhances renal and metabolic protection but also optimizes the safety tolerability profile for DKD patients.

### 4.5 Clinical implications and recommendations

Based on the GRADE evidence grading system, the following stratified treatment recommendations are proposed:

For patients with rapid decline in renal function (manifested by elevated serum creatinine) accompanied by significant systemic inflammatory response, it is recommended to prioritize the treatment regimen of Bailing Capsules combined with sodium-glucose cotransporter two inhibitors (SGLT-2i), aiming to delay the progression of end-stage renal disease (ESKD) to the greatest extent.

For patients with a large amount of proteinuria, immune inflammatory damage mediated by interleukin-6 (IL-6), poor glycemic control, or significant edema as the main manifestations, it is recommended to prioritize the treatment regimen of Huangkui Capsules combined with SGLT-2i, with the focus on repairing the glomerular filtration barrier and strengthening metabolic regulation.

Although the existing evidence initially shows the trend differences in stratified treatment, the evidence quality of some indicators is low (Low certainty). More direct comparative studies are still needed in the future to further verify its specific anti-inflammatory mechanism.

### 4.6 Advantages and limitations

The advantage of this study lies in the fact that it encompasses 11 comprehensive observation indicators and attempts for the first time to explain the therapeutic efficacy differences observed clinically from a mechanistic perspective (i.e., at the level of inflammatory factor targets), thereby forming a relatively complete chain of evidence. Additionally, to ensure the reliability of the analysis results, we were relatively strict in the data screening stage and excluded seven studies that had reporting errors or extreme values in key indicators. Although this resulted in a loss of some initial sample size, it did enhance the internal validity of the analysis and allowed the final conclusion to be based on more consistent and reliable evidence. Conversely, this also highlights the necessity of standardized data reporting in future original studies.

However, this study also has several limitations. (1) The number of original studies that included some mechanism-related indicators (such as IL-6) was not large (only 8), resulting in a wider confidence interval for the statistical results, but it did not affect the judgment of the overall trend. (2) Although a significant proportion of the included primary studies lacked detailed reporting on blinding and allocation concealment, introducing potential performance and detection biases, it is crucial to note that the primary efficacy endpoints in this meta-analysis (such as Scr, BUN, and 24 h-UTP) are objective laboratory measurements. These objective parameters are intrinsically less susceptible to subjective bias compared to patient-reported outcomes, thereby preserving the reliability of the pooled estimates. (3) The included research subjects were all from the Chinese population, and the conclusion should be cautious when extrapolated. (4) Currently, there is a lack of long-term follow-up data of more than 1 year for further verification. (5) Furthermore, a rigorous assessment using the ConPhyMP tool revealed a systematic reporting deficiency across the included primary RCTs. Specifically, these clinical studies uniformly lack detailed reporting on commercial product characteristics (such as specific batch numbers) and fail to provide quantitative phytochemical profiling or analytical chromatograms for the specific batches of CCPPs (Bailing Preparations and Huangkui Capsules) administered to patients. This highlights a critical need for enhanced reporting standards in future clinical trials involving botanical drugs.

## 5 Conclusion

The combination of Bailing preparation and Huangkui capsule with SGLT-2i in the treatment of DKD has shown definite efficacy. However, there are significant differences in their mechanisms of action, and it is not a simple case of homogeneity selection. The Bailing preparation focuses on systemic anti-inflammatory effects and overall kidney function protection, while the Huangkui capsule is more focused on local anti-inflammatory effects, hypoglycemic effects, and reduction of proteinuria. They have different positions and complementary values in clinical applications. This finding provides robust evidence-based support for precision and individualized integrated Chinese-Biomedicine treatment of DKD. Clinical selection should be tailored to patients’ phenotypic characteristics (creatinine-dominant vs proteinuria-dominant).
