# Supplementary material for: Differential efficacy and anti-inflammatory mechanisms of Bailing Preparations versus Huangkui Capsules combined with SGLT-2 inhibitors for diabetic kidney disease: a network meta-analysis and GRADE assessment
Source: Front Pharmacol. 2026 May 29;17:1812118. doi: 10.3389/fphar.2026.1812118 (PMC13260605; doi:10.3389/fphar.2026.1812118)
Supplement: Supplementary file 1 [file DataSheet1.zip › 补充/24UP两两对比森林图.pdf]

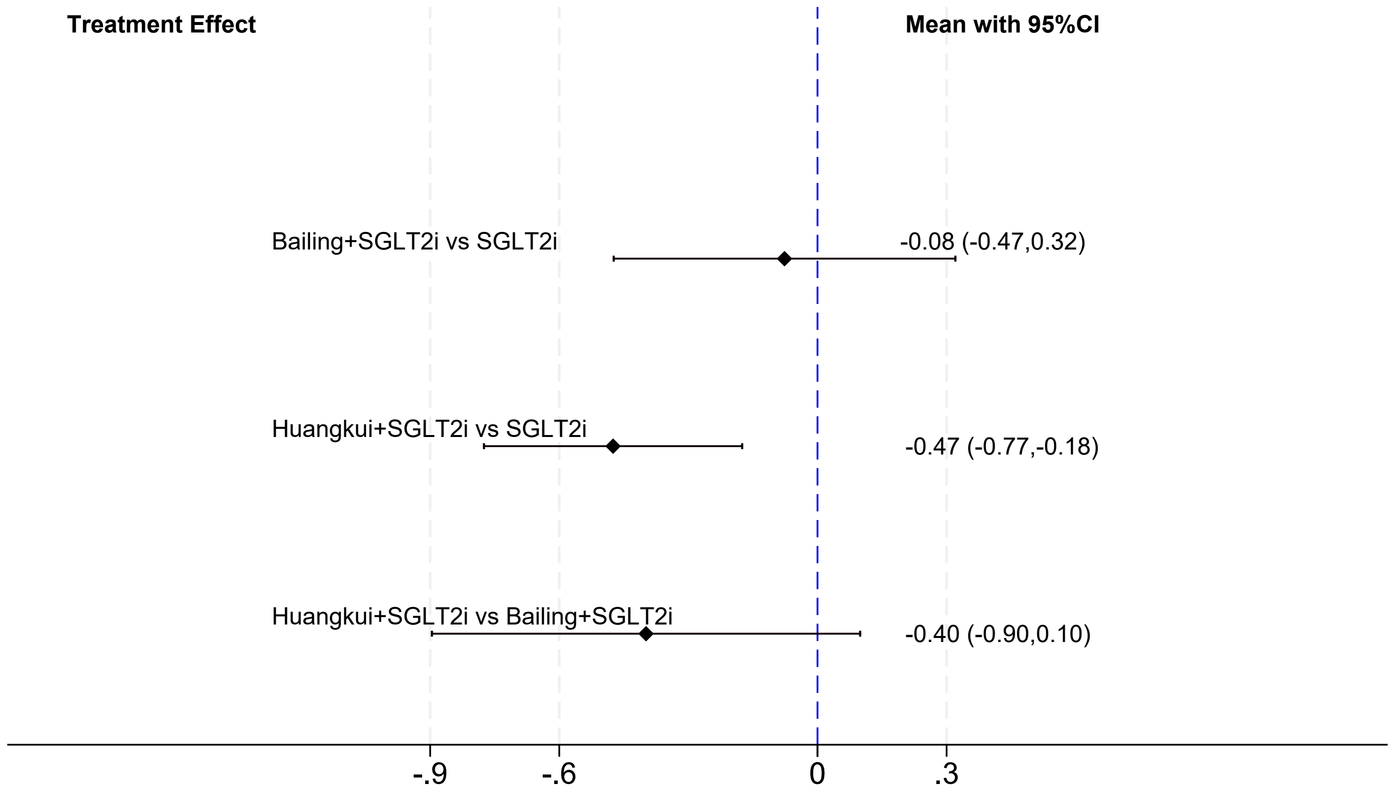

Treatment Effect
Mean with 95%CI
Bailing+SGLT2i vs SGLT2i
-0.08 (-0.47,0.32)
Huangkui+SGLT2i vs SGLT2i
-0.47 (-0.77,-0.18)
Huangkui+SGLT2i vs Bailing+SGLT2i
-0.40 (-0.90,0.10)
-.9
-.6
0
.3
