# Supplementary material for: Differential efficacy and anti-inflammatory mechanisms of Bailing Preparations versus Huangkui Capsules combined with SGLT-2 inhibitors for diabetic kidney disease: a network meta-analysis and GRADE assessment
Source: Front Pharmacol. 2026 May 29;17:1812118. doi: 10.3389/fphar.2026.1812118 (PMC13260605; doi:10.3389/fphar.2026.1812118)
Supplement: Supplementary file 1 [file DataSheet1.zip › 补充/2hPG两两对比森林图.pdf]

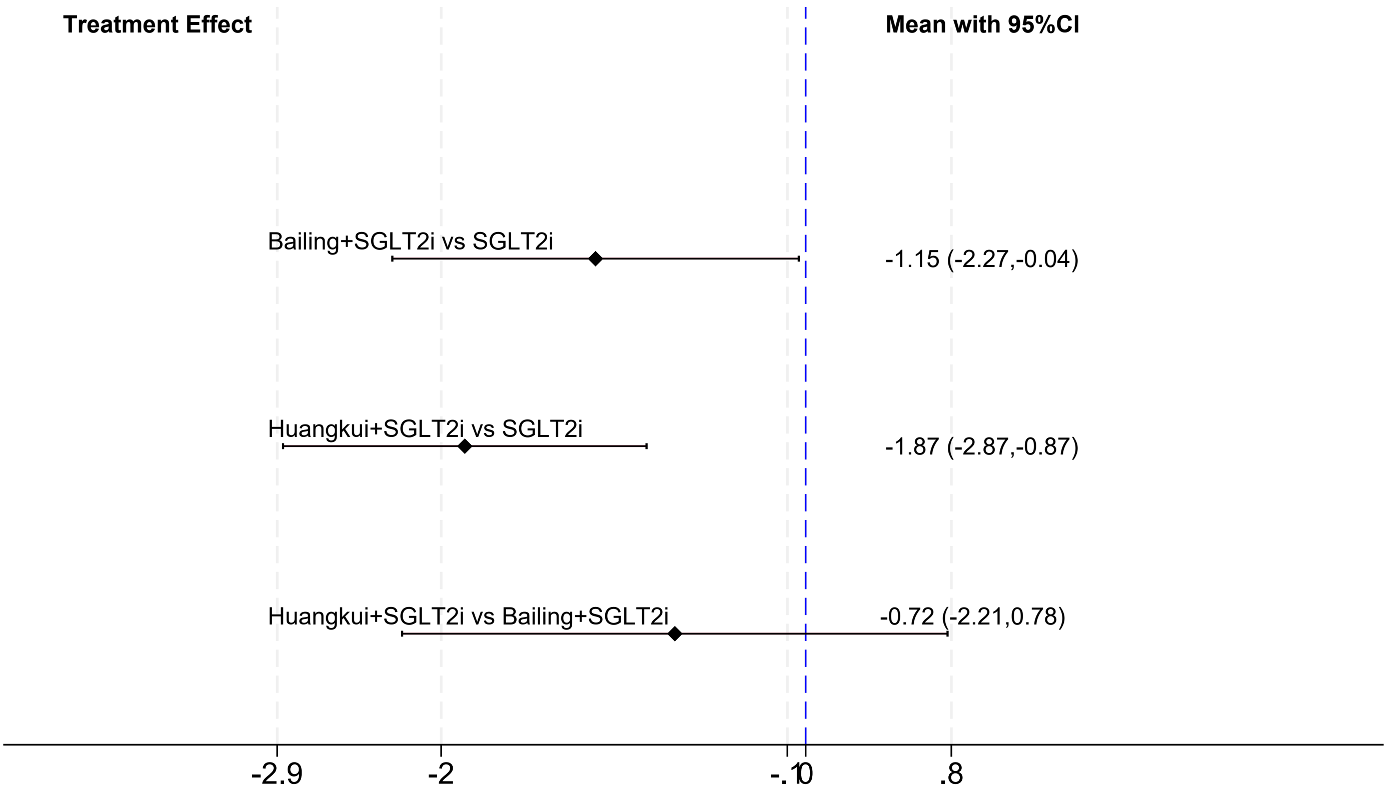
Treatment Effect
Mean with 95%CI
Bailing+SGLT2i vs SGLT2i
-1.15 (-2.27,-0.04)
Huangkui+SGLT2i vs SGLT2i
-1.87 (-2.87,-0.87)
Huangkui+SGLT2i vs Bailing+SGLT2i
-0.72 (-2.21,0.78)
-2.9
-2
-.1
0
.8
