# Supplementary material for: Differential efficacy and anti-inflammatory mechanisms of Bailing Preparations versus Huangkui Capsules combined with SGLT-2 inhibitors for diabetic kidney disease: a network meta-analysis and GRADE assessment
Source: Front Pharmacol. 2026 May 29;17:1812118. doi: 10.3389/fphar.2026.1812118 (PMC13260605; doi:10.3389/fphar.2026.1812118)
Supplement: Supplementary file 1 [file DataSheet1.zip › 补充/FPG两两对比森林图.pdf]

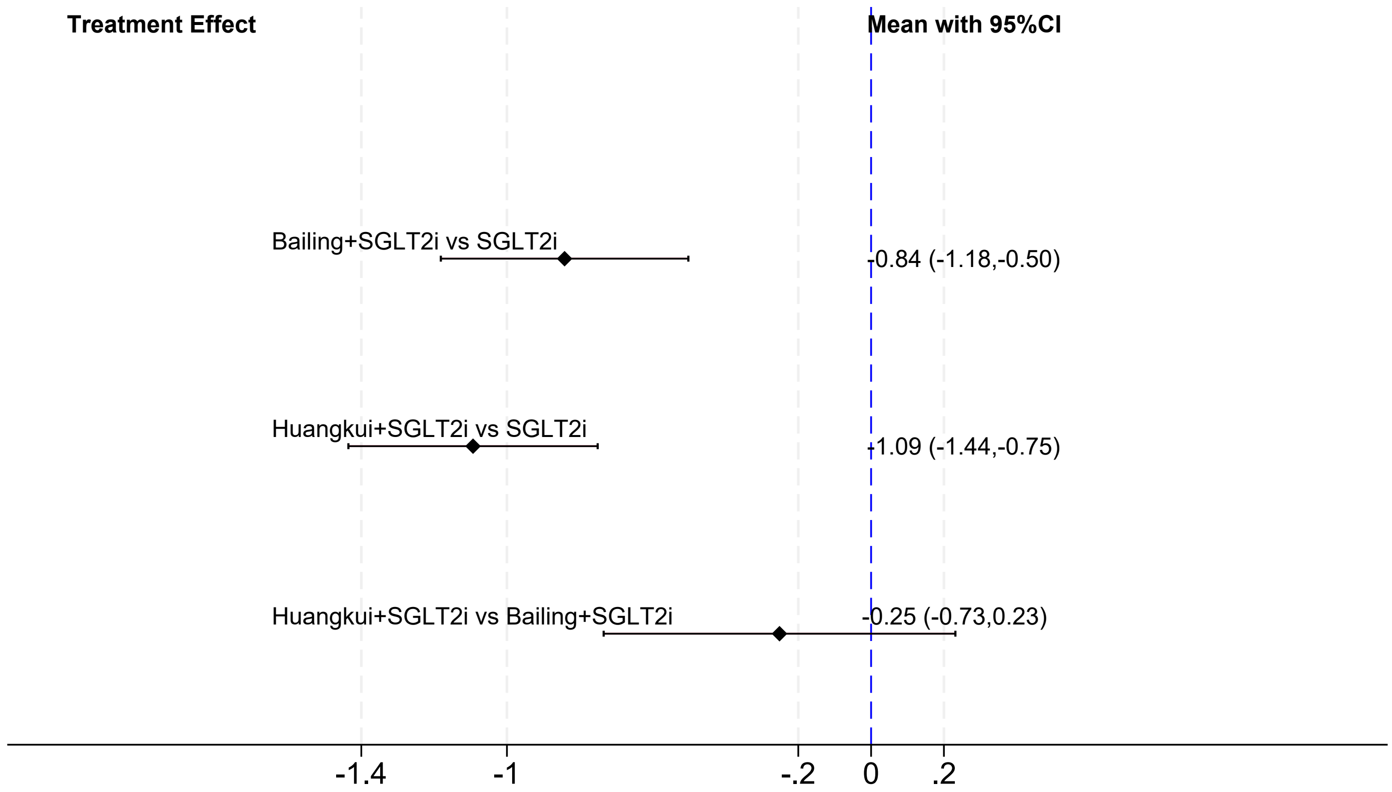
Treatment Effect
Mean with 95%CI
Bailing+SGLT2i vs SGLT2i
-0.84 (-1.18,-0.50)
Huangkui+SGLT2i vs SGLT2i
-1.09 (-1.44,-0.75)
Huangkui+SGLT2i vs Bailing+SGLT2i
-0.25 (-0.73,0.23)
-1.4
-1
-.2
0
.2
