# Supplementary material for: Differential efficacy and anti-inflammatory mechanisms of Bailing Preparations versus Huangkui Capsules combined with SGLT-2 inhibitors for diabetic kidney disease: a network meta-analysis and GRADE assessment
Source: Front Pharmacol. 2026 May 29;17:1812118. doi: 10.3389/fphar.2026.1812118 (PMC13260605; doi:10.3389/fphar.2026.1812118)
Supplement: Supplementary file 1 [file DataSheet1.zip › 补充/SCR两两对比森林图.pdf]

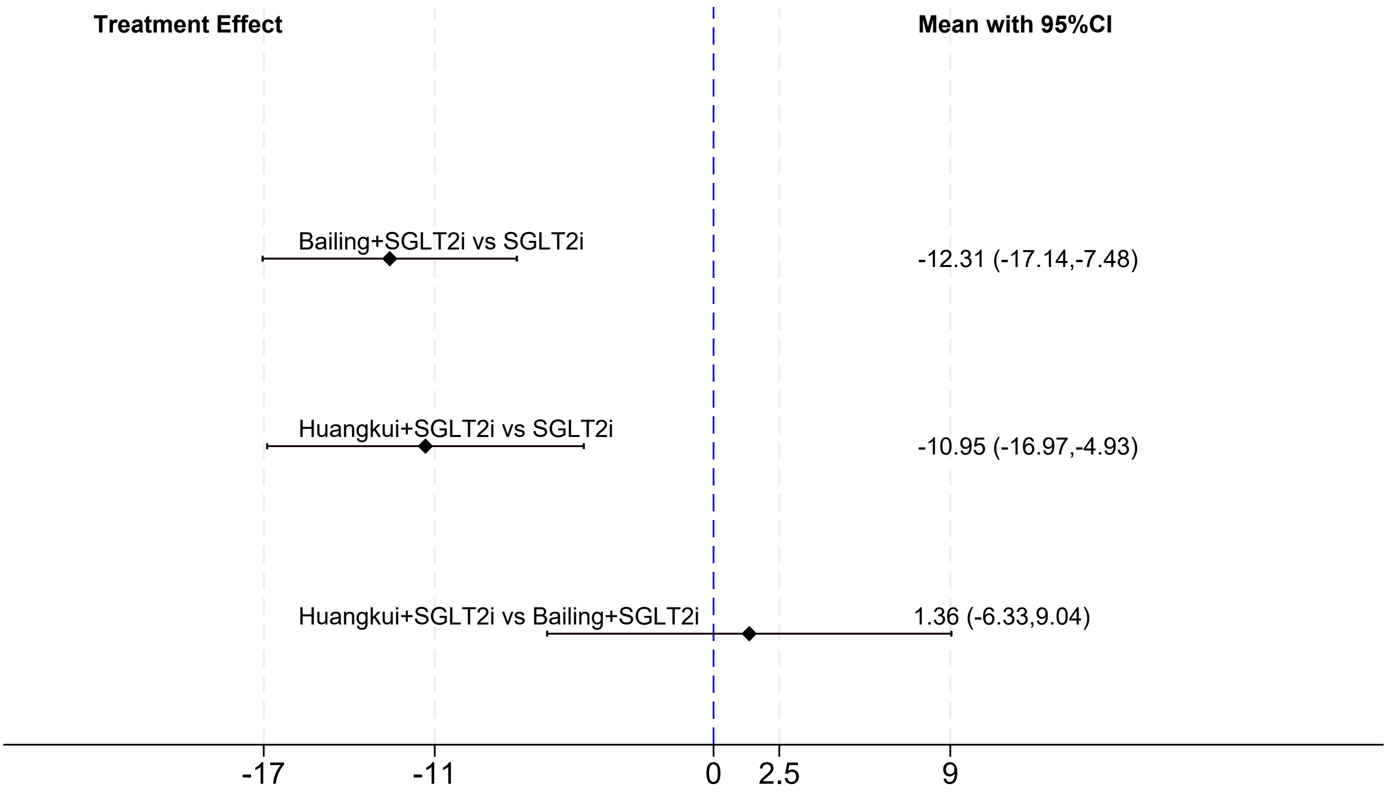
Treatment Effect
Mean with 95%CI
Bailing+SGLT2i vs SGLT2i
-12.31 (-17.14,-7.48)
Huangkui+SGLT2i vs SGLT2i
-10.95 (-16.97,-4.93)
Huangkui+SGLT2i vs Bailing+SGLT2i
1.36 (-6.33,9.04)
-17
-11
0
2.5
9
