# Supplementary material for: Differential efficacy and anti-inflammatory mechanisms of Bailing Preparations versus Huangkui Capsules combined with SGLT-2 inhibitors for diabetic kidney disease: a network meta-analysis and GRADE assessment
Source: Front Pharmacol. 2026 May 29;17:1812118. doi: 10.3389/fphar.2026.1812118 (PMC13260605; doi:10.3389/fphar.2026.1812118)
Supplement: Supplementary file 1 [file DataSheet1.zip › 补充/SCR漏斗图.pdf]

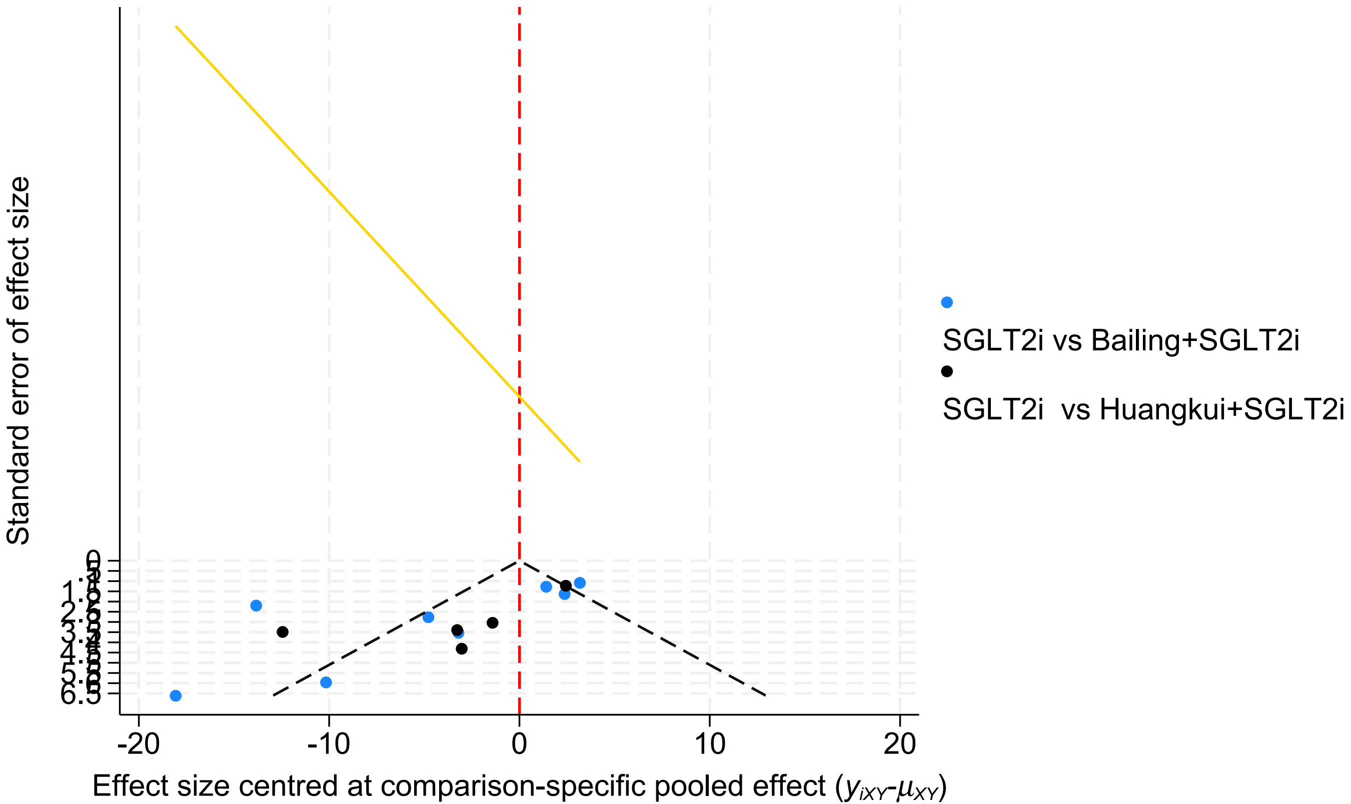

Standard error of effect size
0
6.5
-20
-10
0
10
20
Effect size centred at comparison-specific pooled effect ($y_{iXY}$-$\mu_{XY}$)
SGLT2i vs Bailing+SGLT2i
SGLT2i vs Huangkui+SGLT2i
