# Supplementary material for: Differential efficacy and anti-inflammatory mechanisms of Bailing Preparations versus Huangkui Capsules combined with SGLT-2 inhibitors for diabetic kidney disease: a network meta-analysis and GRADE assessment
Source: Front Pharmacol. 2026 May 29;17:1812118. doi: 10.3389/fphar.2026.1812118 (PMC13260605; doi:10.3389/fphar.2026.1812118)
Supplement: Supplementary file 1 [file DataSheet1.zip › 补充/TNF-α两两对比森林图.pdf]

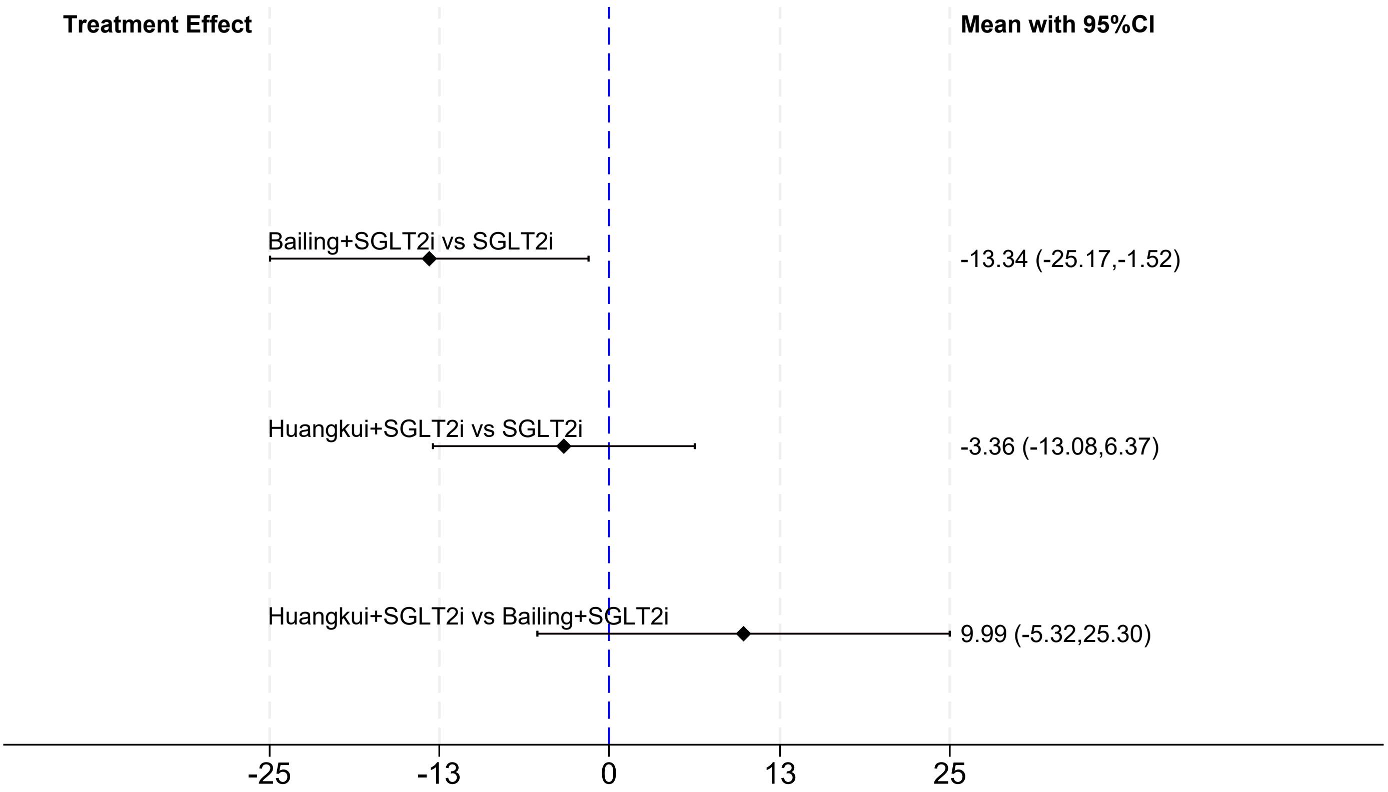

Treatment Effect
Mean with 95%CI
Bailing+SGLT2i vs SGLT2i
-13.34 (-25.17,-1.52)
Huangkui+SGLT2i vs SGLT2i
-3.36 (-13.08,6.37)
Huangkui+SGLT2i vs Bailing+SGLT2i
9.99 (-5.32,25.30)
-25
-13
0
13
25
