# Supplementary material for: Differential efficacy and anti-inflammatory mechanisms of Bailing Preparations versus Huangkui Capsules combined with SGLT-2 inhibitors for diabetic kidney disease: a network meta-analysis and GRADE assessment
Source: Front Pharmacol. 2026 May 29;17:1812118. doi: 10.3389/fphar.2026.1812118 (PMC13260605; doi:10.3389/fphar.2026.1812118)
Supplement: Supplementary file 1 [file DataSheet1.zip › 补充/不良反应两两对比森林图.pdf]

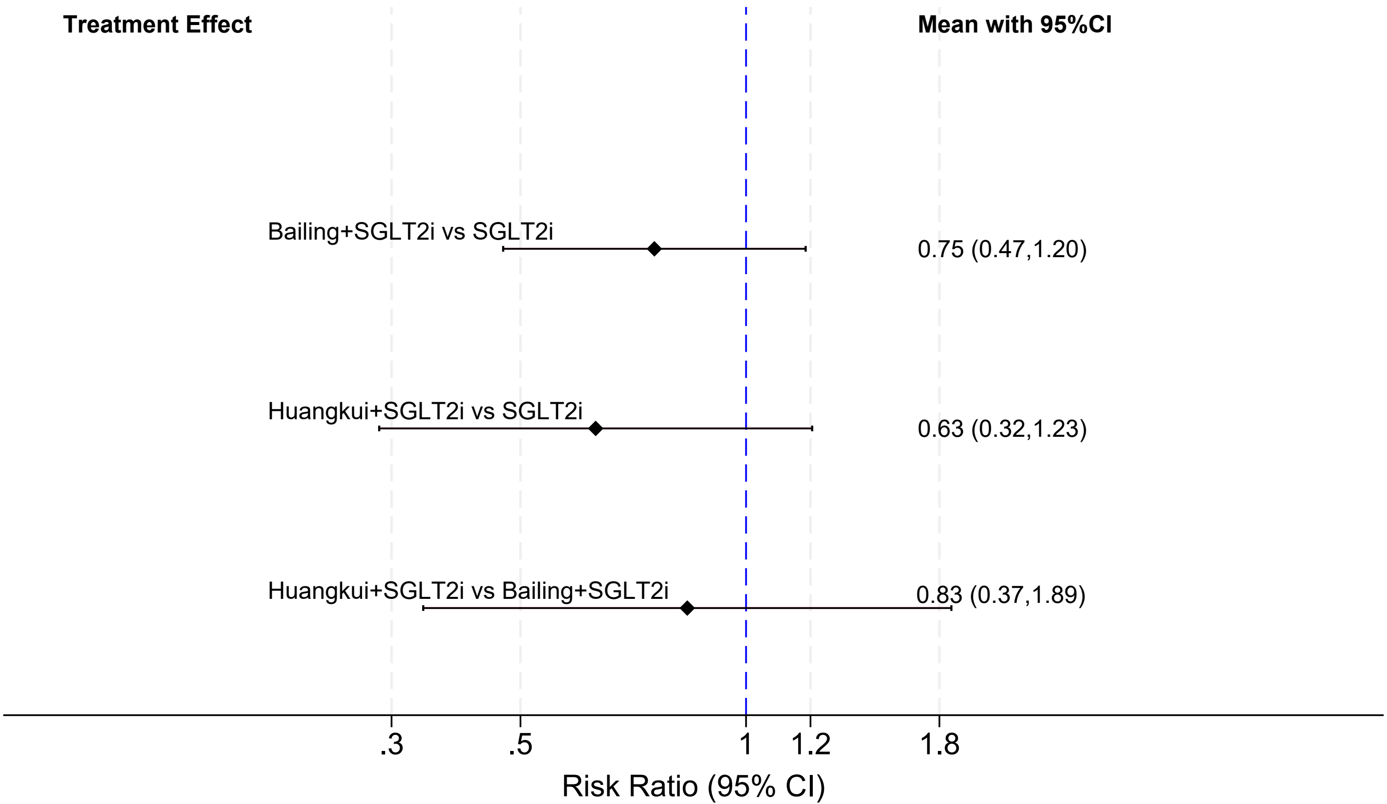
Treatment Effect
Mean with 95%CI
Bailing+SGLT2i vs SGLT2i
0.75 (0.47,1.20)
Huangkui+SGLT2i vs SGLT2i
0.63 (0.32,1.23)
Huangkui+SGLT2i vs Bailing+SGLT2i
0.83 (0.37,1.89)
.3
.5
1
1.2
1.8
Risk Ratio (95% CI)
