# Supplementary figures and images for: Differential efficacy and anti-inflammatory mechanisms of Bailing Preparations versus Huangkui Capsules combined with SGLT-2 inhibitors for diabetic kidney disease: a network meta-analysis and GRADE assessment
Source: Front Pharmacol. 2026 May 29;17:1812118. doi: 10.3389/fphar.2026.1812118 (PMC13260605; doi:10.3389/fphar.2026.1812118)

Standard error of effect size

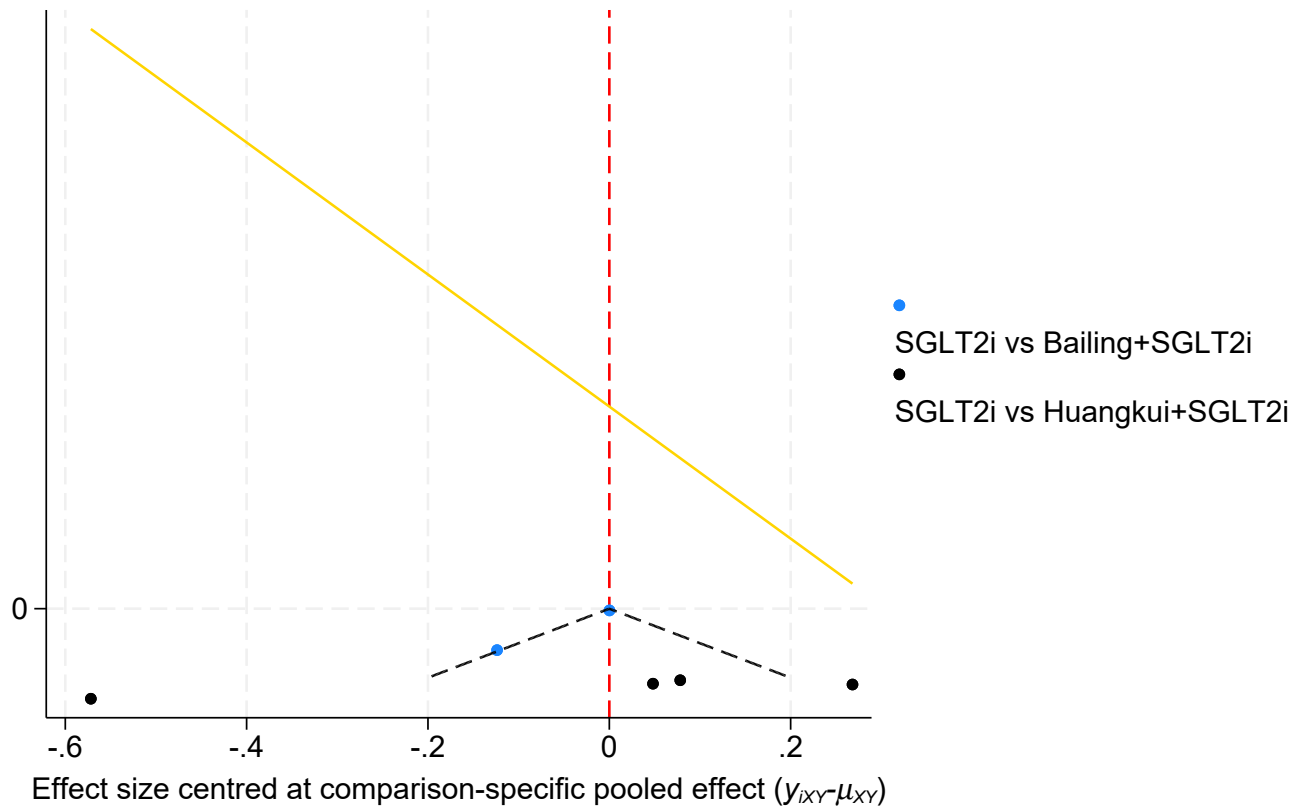

Supplement: Supplementary file 1 [file DataSheet1.zip › 补充/24UP漏斗图.pdf]

Standard error of effect size

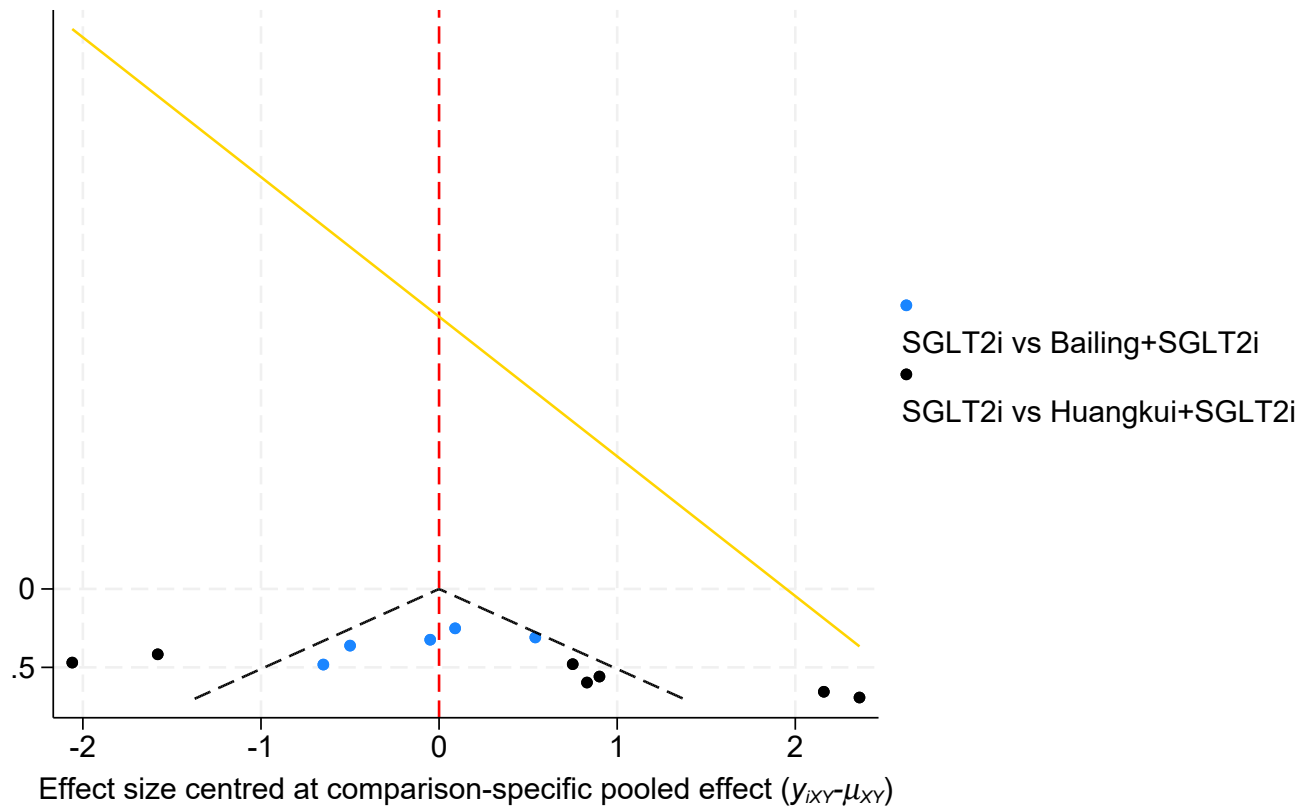

Supplement: Supplementary file 1 [file DataSheet1.zip › 补充/2hPG漏斗图.pdf]

Standard error of effect size

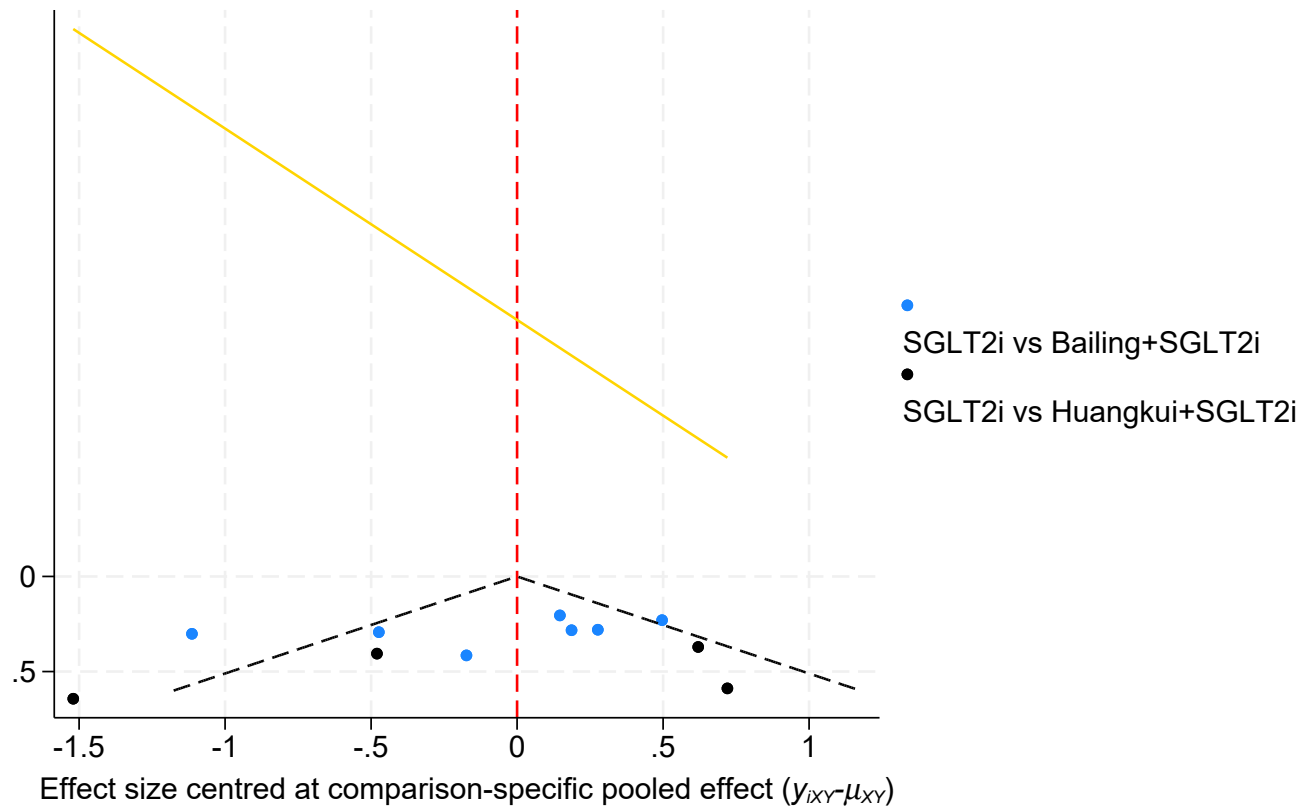

Supplement: Supplementary file 1 [file DataSheet1.zip › 补充/BUN漏斗图.pdf]

Standard error of effect size

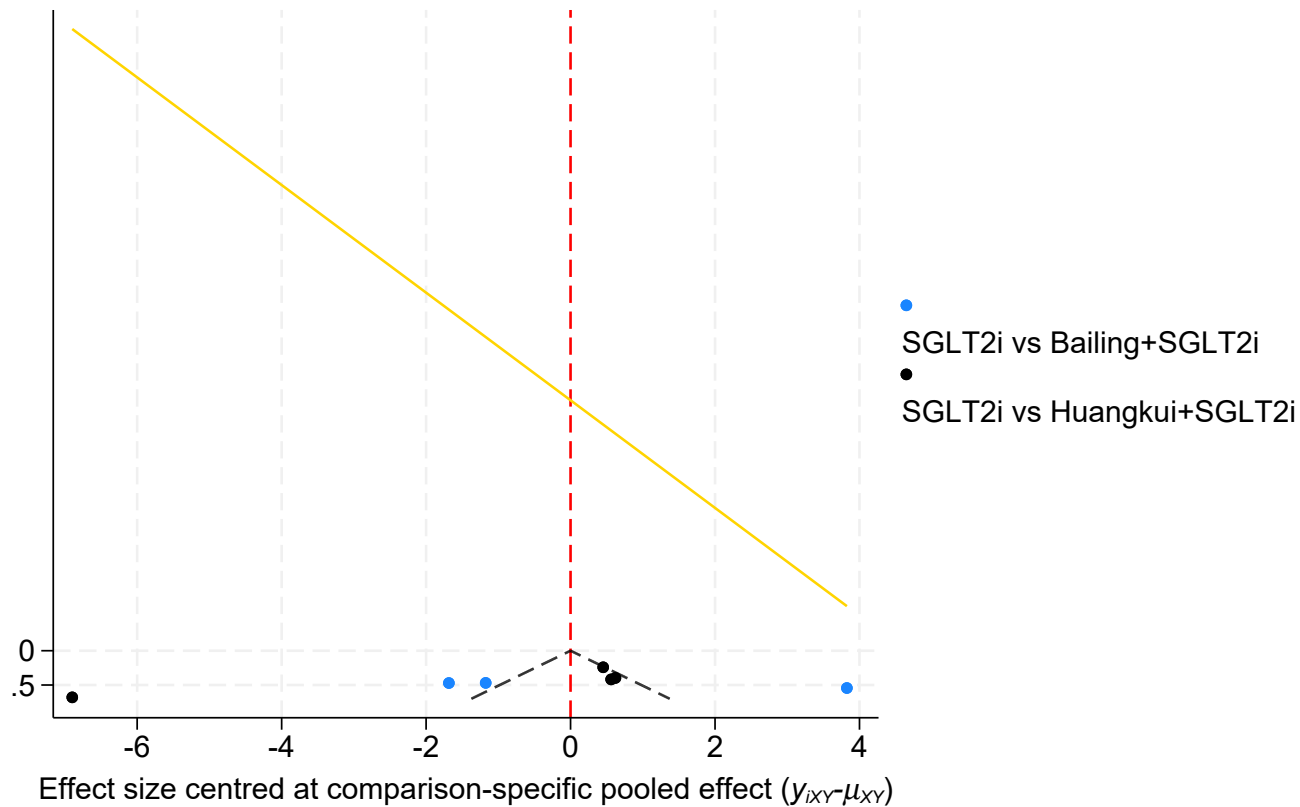

Supplement: Supplementary file 1 [file DataSheet1.zip › 补充/CPR漏斗图.pdf]

Standard error of effect size

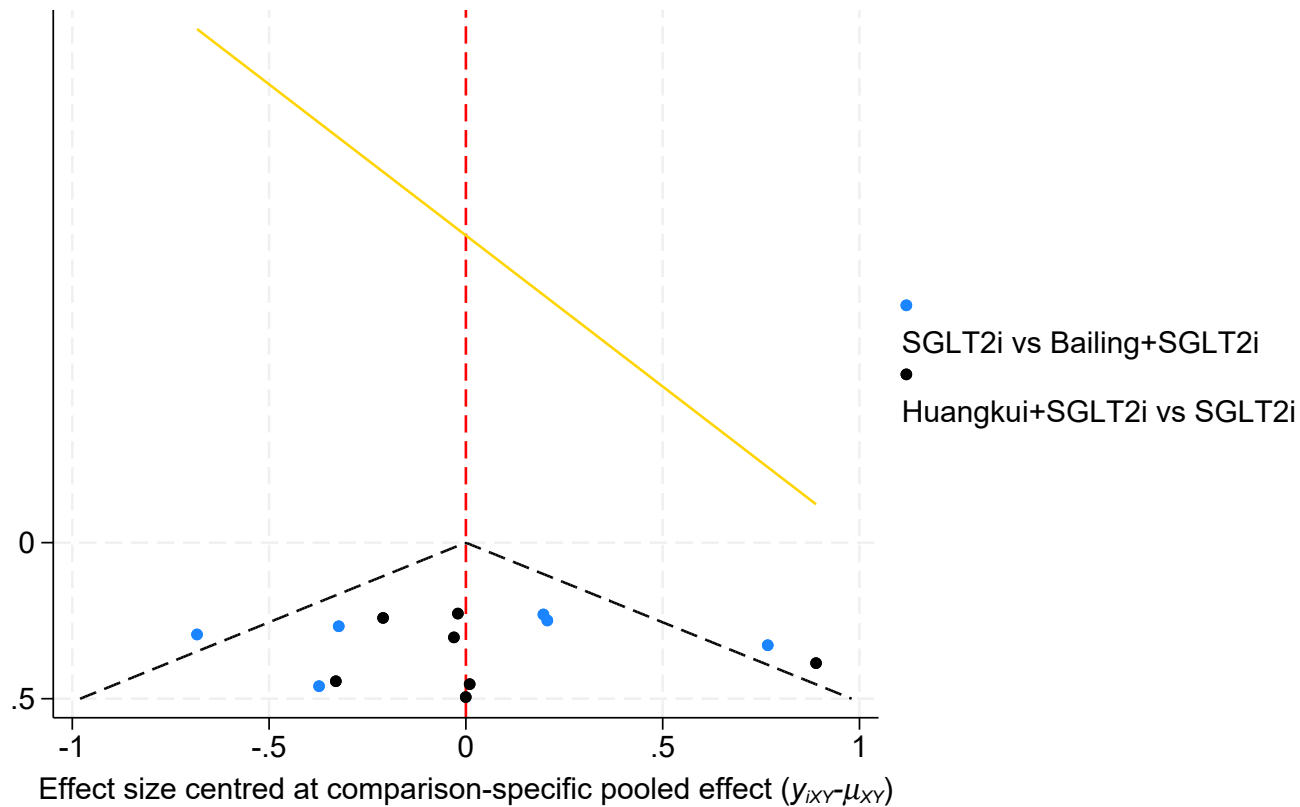

Supplement: Supplementary file 1 [file DataSheet1.zip › 补充/FPG漏斗图.pdf]

Standard error of effect size

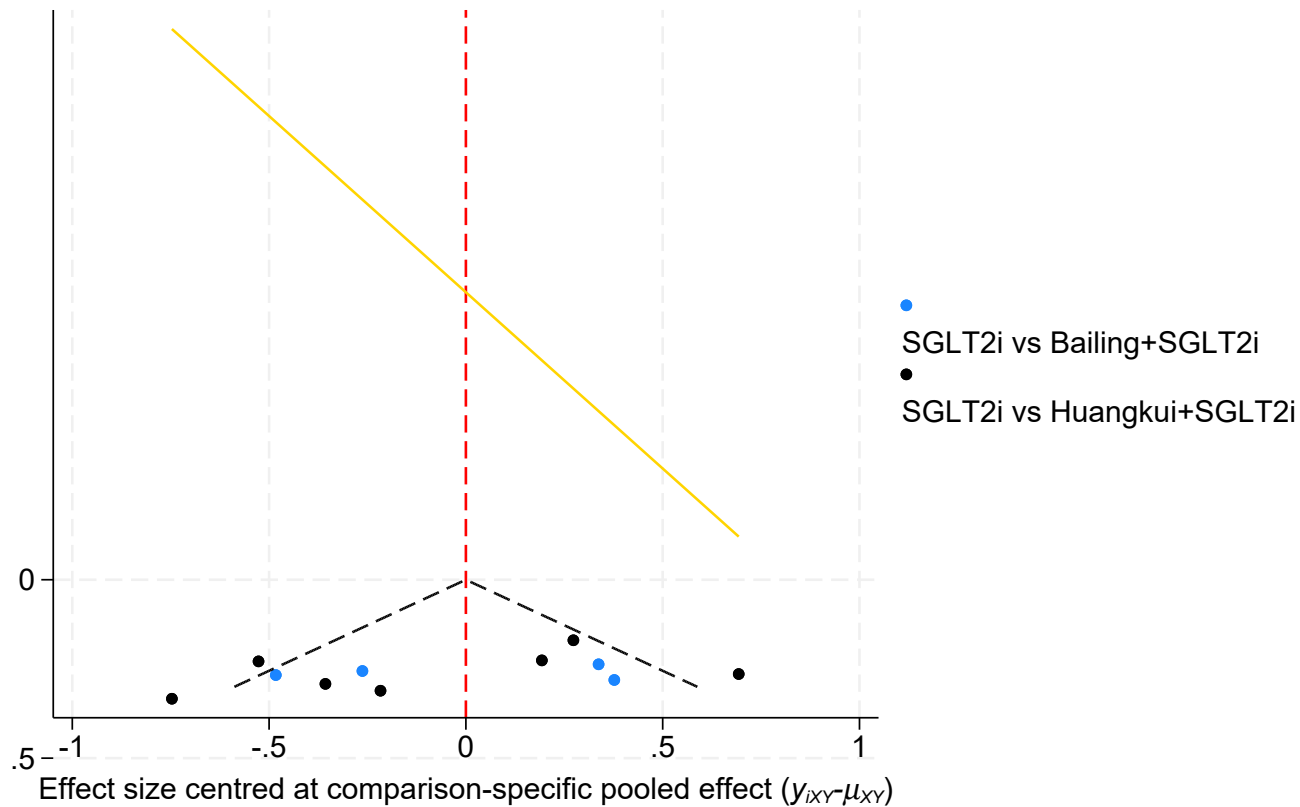

Supplement: Supplementary file 1 [file DataSheet1.zip › 补充/HbAlc漏斗图.pdf]

Treatment Effect

Mean with 95%CI

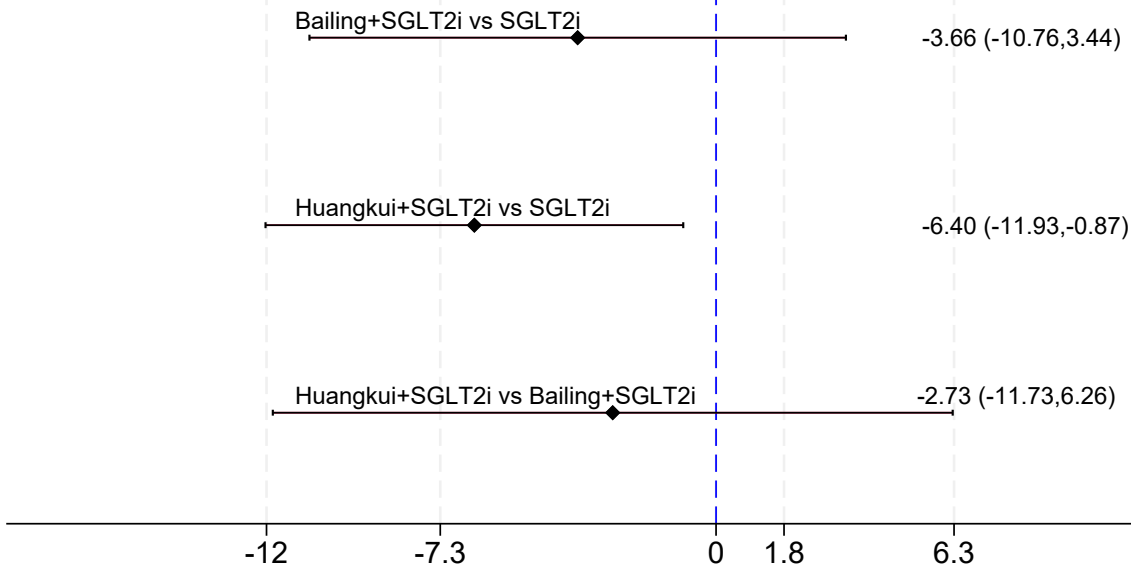

Supplement: Supplementary file 1 [file DataSheet1.zip › 补充/IL-6两两对比森林图.pdf]

Standard error of effect size

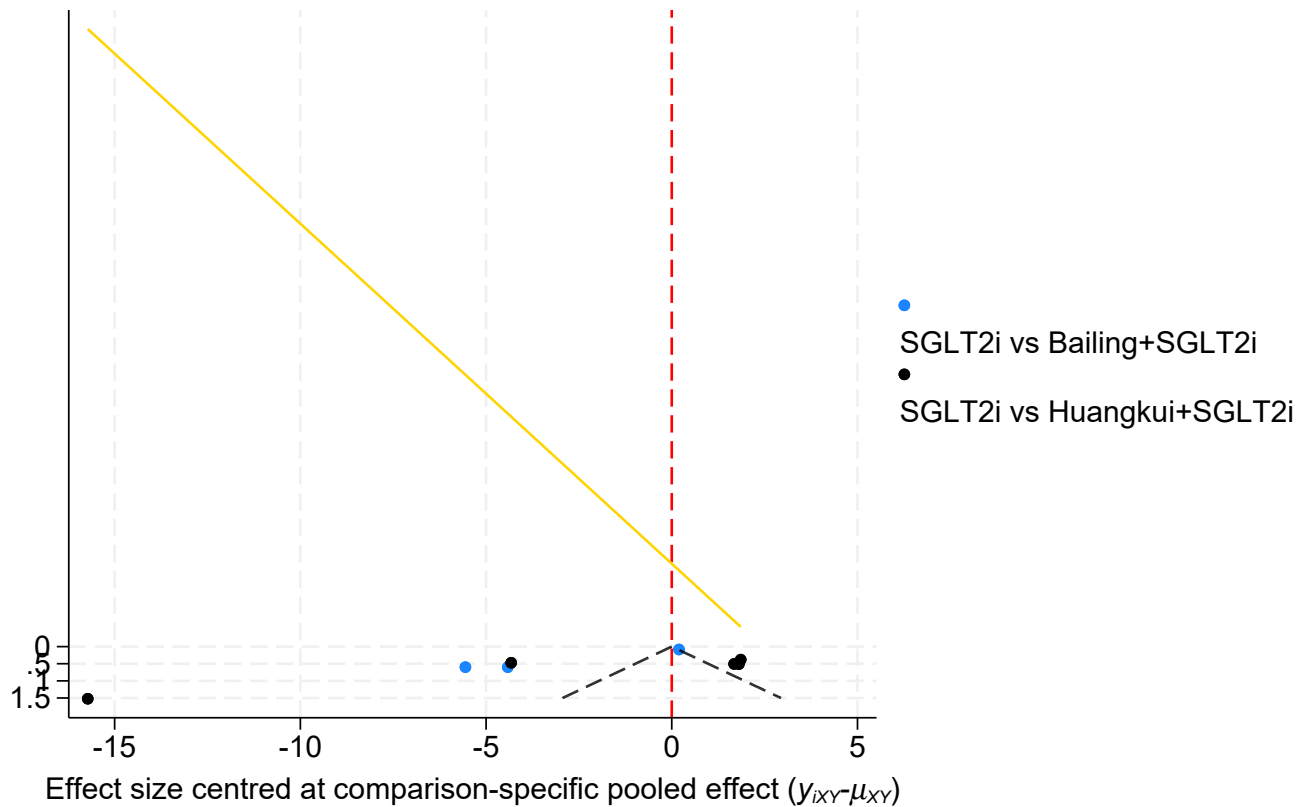

Supplement: Supplementary file 1 [file DataSheet1.zip › 补充/IL-6漏斗图.pdf]

Standard error of effect size

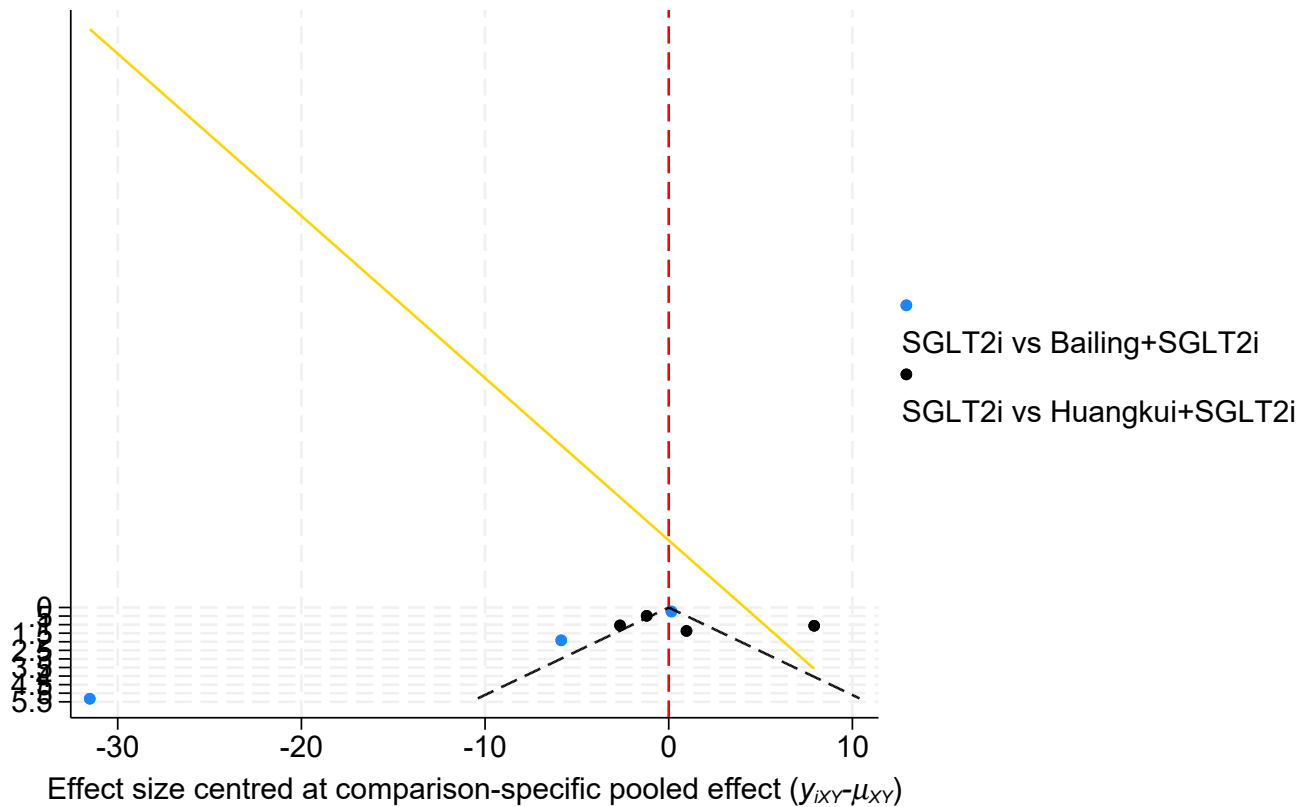

Supplement: Supplementary file 1 [file DataSheet1.zip › 补充/TNF-α漏斗图.pdf]

Standard error of effect size

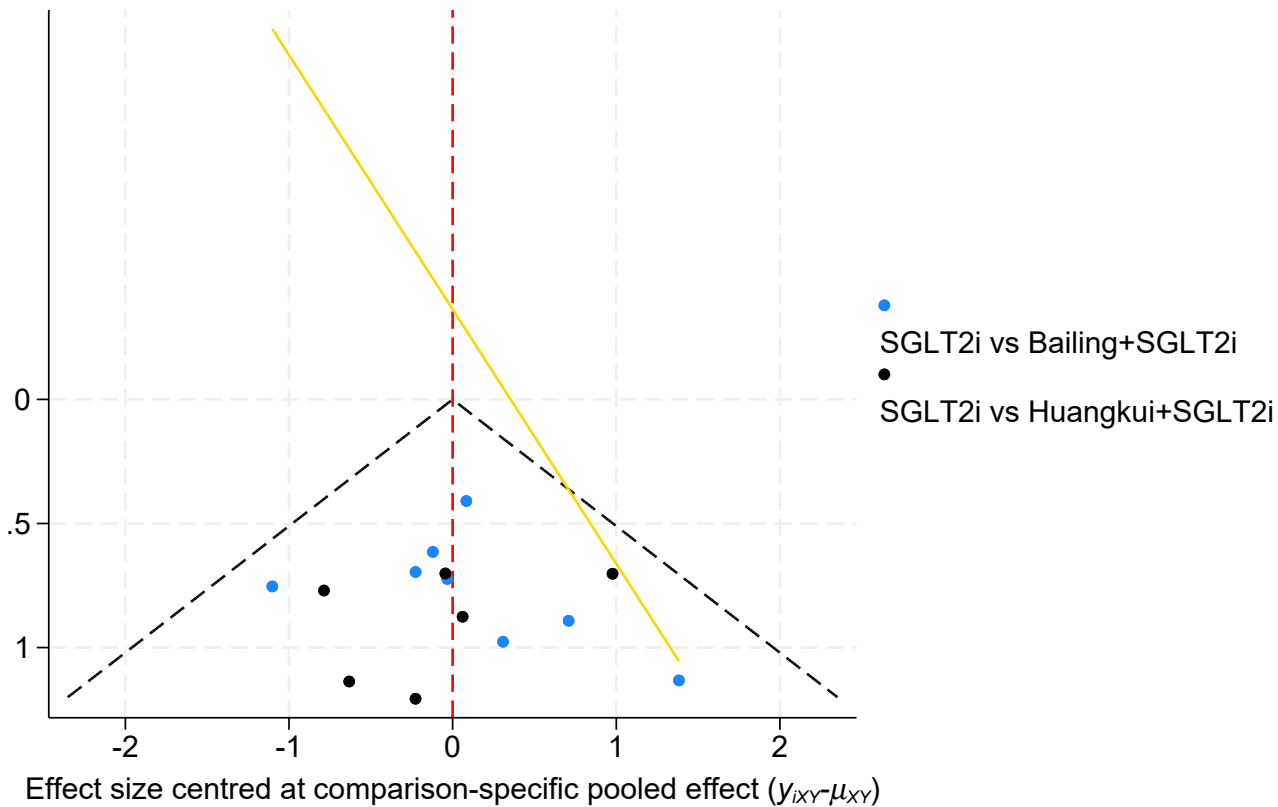

Supplement: Supplementary file 1 [file DataSheet1.zip › 补充/不良反应漏斗图.pdf]

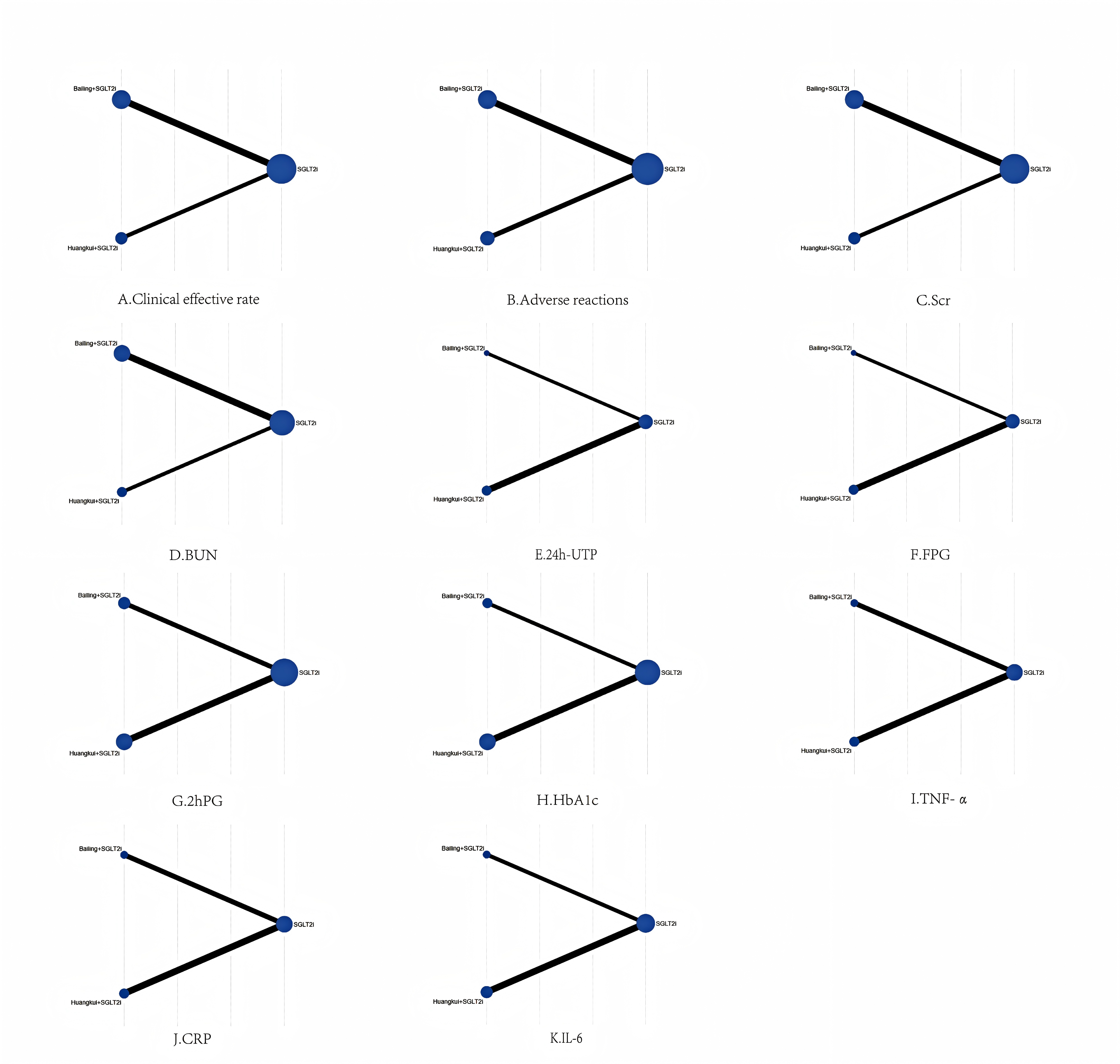

Supplement: Supplementary file 1 [file DataSheet1.zip › 补充/网状拼图.png]
